# Registered Report: Replication and Extension of Nozaradan, Peretz, Missal and Mouraux (2011)

**DOI:** 10.1101/2025.03.13.643168

**Published:** 2025-03-17

**Authors:** Karli M. Nave, Erin E. Hannon, Joel S. Snyder

**Keywords:** frequency tagging, auditory neuroscience, music cognition, beat and meter perception, replication, multilab, preregistration

## Abstract

Cognitive neuroscience research has attempted to disentangle stimulus-driven processing from conscious perceptual processing for decades. Some prior evidence for neural processing of perceived musical beat (periodic pulse) may be confounded by stimulus-driven neural activity. However, one study used frequency tagging, which measures electrical brain activity at frequencies present in a stimulus, to show increased brain activity at imagery-related frequencies when listeners imagined a metrical pattern while listening to an isochronous auditory stimulus (Nozaradan et al., 2011) in a manner that controlled for stimulus factors. It is unclear though whether this represents repeatable evidence for conscious perception of beat and whether the effect is influenced by relevant music experience, such as music and dance training. This registered report details the results of 13 independent conceptual replications of Nozaradan et al. (2011), all using the same vetted protocol. Listeners performed the same imagery tasks as in Nozaradan et al. (2011), with the addition of a behavioral task on each trial to measure conscious perception. Meta-analyses examined the effect of imagery condition, revealing smaller raw effect sizes (Binary: 0.03 μV, Ternary: 0.03 μV) than in the original study (Binary: 0.12 μV, Ternary: 0.20 μV) with no moderating effects of music or dance training. The difference in estimated effects sizes (this study: n = 152, *η*_*p*_^*2*^ =.03 - .04; 2011 study: n = 8, *η*_*p*_^*2*^ =.62 - .76) suggests that large sample sizes may be required to reliably observe these effects, which challenges the use of frequency tagging as a method to study (neural correlates of) beat perception. Furthermore, a binary logistic regression on individual trials revealed that only neural activity at the stimulus frequency predicted performance on the imagery-related task; contrary to our hypothesis, the neural activity at the imagery-related frequency was not a significant predictor. We discuss possible explanations for discrepancies between these findings and the original study and implications of the extensions provided by this registered report.

## 1 Introduction

In cognitive neuroscience research, a pervasive problem is disentangling neural activity that reflects the processing of stimulus features from activity that reflects conscious experience and other high-level processes. Much research has been devoted to solving this problem in several areas of cognitive science, including visual mental representation (Kosslyn, Ball, & Reiser, 1978; Z. W. Pylyshyn, 1981), speech perception (e.g., Dehaene-Lambertz et al., 2005) auditory spatial orienting (e.g., Rosen et al., 1999), visual and auditory bistable perception (e.g., Pressnitzer & Hupé, 2006), and musical rhythm perception (e.g., Iversen, Repp, & Patel, 2009). Musical *rhythm* refers to a pattern of temporal intervals arranged in a sequence, and musical *beat*^1^ refers to the quasi-isochronous pattern of prominent timepoints that often results from listening to a rhythm (Large & Palmer, 2002). Prior work provided evidence for a neural correlate of conscious beat perception, likely the result of activity in auditory cortical areas. Specifically, the study reported that human listeners had larger neural responses at beat related frequencies than at non-beat-related frequencies in complex rhythms (Nozaradan, Peretz, & Keller, 2016), which was interpreted as evidence for neural correlates of endogenous, top-down musical beat perception on the part of listeners. However, a later study presented the same rhythmic sequences to anesthetized animals and showed that stronger on-beat than off-beat responses could be recorded from the midbrain (Rajendran, Harper, Garcia-Lazaro, Lesica, & Schnupp, 2017), raising the possibility that stimulus-driven exogenous processes, rather than top-down endogenous processes, might drive differential human brain responses to on- versus off-beat events.

Attempts to differentiate exogenous and endogenous processes in the brain are not unique to research on musical beat perception. A related controversy concerns whether mental *imagery* (process of accessing perceptual information from memory) relies on the same or distinct processes as stimulus processing, a debate addressed by studies of visual (Kosslyn et al., 1993, 1978; Z. Pylyshyn, 2003; Z. W. Pylyshyn, 1981) and auditory mental imagery (Halpern & Zatorre, 1999; Zatorre, Halpern, Perry, Meyer, & Evans, 1996). The distinction between bottom-up, stimulus-driven and top-down, percept-driven processes is also evident in research on speech perception. For example, under certain conditions listeners can comprehend a linguistic message when presented with sine-wave speech, even though sine-wave speech has none of the acoustic attributes traditionally assumed to underlie speech perception (e.g., formants, formant transitions, fundamental frequency, etc.) (Remez, Rubin, Pisoni, & Carrell, 1981). The amplitude, latency, and localization of brain responses are different when listeners hear the sine-wave speech as speech than when they hear it as non-speech, even when the same physical stimulus is presented across conditions (Dehaene-Lambertz et al., 2005). Such approaches are particularly promising for distinguishing between stimulus processing and conscious processing because they examine neural responses to the same physical stimulus across conditions in which the perception of that stimulus is altered, either by context or task goals.

Musical beat is an excellent candidate for examining the distinction between stimulus-driven and perception-related electrophysiological responses in the auditory central nervous system because its regularity is so prominent to the music listener, and it does not always depend on continuous physical input. For some musical rhythms, two or more different yet valid interpretations of the musical beat pattern can be heard, as with other auditory and visual bistable stimuli (Iversen et al., 2009; Kim & Blake, 2005; Pressnitzer & Hupé, 2006). This allows for more confident conclusions to be drawn about the higher-level processes involved in perception. Research on rhythm perception has indeed revealed brain activity that is to some degree isomorphic with the beat, as measured using both electroencephalography (EEG) and magnetoencephalography (MEG) (Fujioka, Trainor, Large, & Ross, 2009; Nozaradan et al., 2011; Snyder & Large, 2005; Zanto, Snyder, & Large, 2006). According to the Dynamic Attending Theory, listeners’ attention fluctuates at frequencies isomorphic with rhythmic external stimuli, causing the brain to form temporal expectancies about incoming auditory information (Jones & Boltz, 1989; Large & Jones, 1999). Similarly, Predictive Coding Theory posits that the human brain forms predictions based on the probability of a recurring pattern of events, such as musical beats in an auditory stimulus, specifically with the goal of minimizing prediction error (Vuust & Witek, 2014). Auditory rhythms are presumed to not only lead to entrainment at stimulus-related frequencies in the brain activity, but also to entrainment at perception-related frequencies in the brain. However, it is not entirely clear whether these previously discovered neural markers of auditory rhythm processing truly index perception of beat and meter, or whether they simply reflect stimulus processing that is propagated from low-level to high-level areas of the brain. Recent evidence suggests that that there may be significant contributions from low-level auditory brain areas that give rise to the perception of musical beat, including scalp-recorded activity that primarily arises from the brainstem (Tierney & Kraus, 2013) and more direct recording of action potential firing rates from midbrain neurons (Rajendran et al., 2017).

Although studies have attempted to disentangle lower-level processes from higher-level processes in musical beat perception, they often confound stimulus features with perception. For example, one study compared EEG responses while participants listened to a rhythm with a beat that was physically present or to a rhythm that required listeners to infer a beat that was not physically present in the stimulus (Nozaradan, Schönwiesner, Keller, Lenc, & Lehmann, 2018). Results showed that the latter beat frequency was evident in cortical brain activity but not in a measure reflecting brainstem activity (the *frequency-following response)*. While this finding was interpreted as supporting the claim that beat-related activity in the brain reflects conscious perception of the beat, physically distinct sound stimuli were used to generate these different brain responses. Several other studies also attempted to highlight differences between perception-related and stimulus-evoked neural processing of musical beat, yet they used different stimuli across conditions (e.g., Fujioka, Trainor, Large, & Ross, 2012; Nozaradan et al., 2016; Winkler, Háden, Ladinig, Sziller, & Honing, 2009).

At the time of pre-registration (2019), only one study had measured cortical responses at musical beat-related frequencies by manipulating the listener perception and holding the stimulus constant. Nozaradan et al. (2011) produced some of the first evidence that brain activity does not solely reflect the physical characteristics of the stimulus but rather listener perception. Listeners imagined a beat pattern (binary or ternary) while listening to a beat-ambiguous auditory stimulus (i.e., a metronome-like sequence of equal amplitude events that could be perceived as having either beat pattern). This study used frequency tagging by transforming the averaged event-related EEG responses from the time domain to the frequency domain and examined the amplitude of brain activity at the beat frequencies. Higher amplitude neural activity was observed at frequencies corresponding to the imagined beat, as compared to non-beat frequencies. These brain responses that were isomorphic to the *imagined* beat frequencies showed an effect of the listener’s imposition of a specific structure onto the stimulus they heard.

This inspired other studies to use the frequency-tagging approach to examine music perception (Celma-Miralles, de Menezes, & Toro, 2016; Chemin, Mouraux, & Nozaradan, 2014; Cirelli, Spinelli, Nozaradan, & Trainor, 2016; Nozaradan, Peretz, & Mouraux, 2012; Stupacher, Wood, & Witte, 2017; Tal et al., 2017; Tierney & Kraus, 2015). Frequency-tagging has also been used to study perception in other areas of auditory research, such as word, phrase, and sentence level comprehension of speech (e.g., Ding, Melloni, Zhang, Tian, & Poeppel, 2016) and to study the effect of attention to targets during auditory scene analysis (e.g., Elhilali, Xiang, Shamma, & Simon, 2009). Earlier work used this technique in the visual domain to study visual bistable perception (e.g., Silberstein, 1995; Tononi, Srinivasan, Russell, & Edelman, 1998). Despite the breadth of research using this technique, there is little evidence, aside from the 2011 paper, indicating that frequency-related cortical brain responses reflect the listener’s perception of the beat in music, rather than low-level stimulus properties.

Furthermore, it is unclear to what extent this change in brain response at the beat frequency is influenced by other factors, such as the music training and dance training of the listener. Prior work has suggested that not only are high-level cortical auditory evoked potentials more reflective of beat and meter in rhythmically-trained individuals compared to non-rhythmically-trained individuals (Jongsma, Desain, & Honing, 2004), but lower-level responses at the level of the brainstem are enhanced for musicians compared to non-musicians (Parbery-Clark, Skoe, & Kraus, 2009). Musicians are also more sensitive to musical meter (hierarchical organization of strong and weak beats) than non-musicians (Palmer & Krumhansl, 1990). In addition, dancers exhibit enhanced processing of certain aspects of natural music, such as early neural responses to changes in the music when they are relevant to movement (Poikonen, Toiviainen, & Tervaniemi, 2016). To our knowledge, only one study using beat-related frequency-tagging has attempted to address the contribution of musical expertise. In this study, significant beat-related cortical activity was observed for two different types of stimuli: a rhythm with one clear beat pattern (quadruple meter) and a rhythm with a complex pattern comprised of two simultaneous beat patterns (quadruple and triple meters; i.e., a 4:3 polyrhythm) (Stupacher et al., 2017). Both musicians and non-musicians showed significant beat-related activity during the quadruple rhythm and the 4:3 polyrhythm. Interestingly, during a silent period following the rhythm 4:3 polyrhythm, only musicians showed significant beat-related activity, and this activity was correlated with their performance on a beat-matching task that followed the silent period. This suggests that music experience may indeed moderate the processes involved in auditory entrainment to musical rhythms. However, this study did not disentangle whether musical experience influences entrainment processes during auditory rhythm listening; both rhythms contained physical energy at the beat-frequencies themselves, making it impossible to dissociate stimulus-driven activity from perception-driven activity. To date, no one has investigated the extent to which music and dance training influence the perception-related activity demonstrated by Nozaradan et al. (2011). Moreover, given that nearly half of participants in the original 2011 study had extensive musical expertise (15–25 years of music training), it is particularly important to assess perception-driven brain responses in listeners with and without musical expertise.

In the current RR, we conducted a multi-lab study where we conducted a conceptual replication of the above paper, titled “Tagging the neural entrainment to beat and meter” (Nozaradan et al., 2011) and extended the findings to measure 1) the direct relation between the magnitude of the endogenous beat-related brain response and conscious perception of musical beat and 2) the relation between the magnitude of the beat-related brain response and music training. In this project, 14 labs completed independent, pre-registered studies, each using the same RR protocol. A minimum of six labs were required for the project.

The RR had three aims. The first aim was to estimate the true sizes of the effects reported in the original 2011 study by performing meta-analyses across research labs. The study was conducted using methods designed to be as closely matched to the original 2011 study as possible, allowing for an estimate of the meta-analytic replication of the original effects reported. All additional procedures were conducted such that they would not influence the replicated effects. Meta-analytic estimates examined the effect of condition (binary imagery, ternary imagery, or control task) on the amplitude of electrical brain activity in the frequency spectrum at the binary frequency and the ternary frequency, as measured by EEG (outcomes that showed an effect in the original study).^2^ We hypothesized that the true effect size estimates would be similar to those reported by Nozaradan et al. (2011). The second aim of the RR was to improve upon the original methods by collecting a behavioral measure of beat perception on each trial, which allows a closer comparison between perception and brain activity and may additionally enhance listener attention and effort during the imagination task. We hypothesized that performance on the behavioral measure of conscious perception would be predicted by the magnitude of the beat-related brain response. The third and final aim of this RR was to extend our understanding of factors related to the findings of the original 2011 paper by measuring two hypothesized covariates: music training and dance training. We hypothesized that music and dance experience would be related to the magnitude of the beat-related brain response. By measuring years of training for both music and dance, we aimed to account for different types of interaction with music, which varies largely across cultures. The meta-analytic results of these analyses are presented in the results section.

The procedure followed in this RR was specific, unbiased, and transparent. We created a detailed study protocol, including explicit training instructions for the participant tasks, experimental materials, and a detailed experiment set-up guide. We designed a detailed analysis protocol, MATLAB script to process the EEG data, and R scripts to conduct the meta-analyses before viewing the data. Finally, the introduction, methods section, and results section (with placeholders for the final statistical results) were written prior to analyzing the data. All of these materials are publicly available on the Open Science Framework (OSF).

## 2 Disclosures

### 2.1 Preregistration

All preregistered materials are available at the project OSF page: https://osf.io/rpvde/.

### 2.2 Data, materials, and online resources

All materials necessary to conduct the study are available on the OSF project page: https://osf.io/rpvde. Summary data are available on the OSF project page, and raw EEG data are available on OpenNeuro here: https://osf.io/t63gz.

### 2.3 Reporting

We report how we determined our sample size, all data exclusions, all manipulations, and all measures in the study.

### 2.4 Ethical Approval

All participating labs were required to collect their sample of data with the approval of an institutional review board in accordance with the Declaration of Helsinki and provide the protocol number.

## 3 Protocol Development and Requirements

Nave, Hannon, and Snyder proposed this RR project and developed the protocol. The first author of the original study provided the stimulus that they used. The protocol is available on the OSF project page for the RR project (https://osf.io/rpvde/).

*Advanced Methods and Practices in Psychological Science* publicly announced a call for laboratories interested in participating in this RR project on April 23, 2019. The data collection start date was May 1^st^, 2019, although labs were allowed to register to participate and begin data collection any time before the data collection end date. Data collection concluded on April 30^th^, 2022. Initially, we planned for the data collection period to last 12 months (ending April 30^th^, 2020), but due to the COVID-19 pandemic and local requirements to social distance, many labs were unable to resume EEG studies until as late as January 2022.

Prior to conducting the study, each lab submitted a pre-registered plan for implementing the approved protocol, and the first author reviewed each plan to ensure that it met the requirements of the protocol. Summaries of the labs’ pre-registered plans of protocol implementation can be found in Appendix A. Labs were required to note any deviations from the standard protocol, as well as any departures from their pre-registration that occurred during data collection (see Table S1).

## 4 Methods

### 4.1 Participants

Each lab committed to testing a minimum of sixteen^3^ healthy volunteers (after replacing participants who met the exclusion criteria below) between the ages of 18^4^ and 45, with normal hearing and no history of neurological or psychiatric disorders. While we hypothesized that the main analyses conducted in the original study (one-way ANOVAs) may have produced large effect sizes due to music and dance experience of the original sample, we expected that the true effect could be smaller in non-musicians and non-dancers. An a priori power analysis using G*Power 3.1 revealed that each lab would need 15 participants to detect a medium effect size (η^2^ = .06) at .80 power (other parameters: alpha = .05, number of conditions = 3, number of repeated measures = 10, correlation among repeated measures = 0.5, non-sphericity correction = 1). Thus, we required 16 participants for a full lab sample to allow us to detect a medium to large effect size in order to capture a true effect that is smaller in size from the original study (η^2^ = .62, η^2^ = .76).

Of the 18 labs that applied to participate, 15 labs collected data for this study, and 14 labs completed the entire study protocol. Lab 14 was removed from any subsequent analyses after participant exclusions revealed a final sample that was too small (*n* = 4; minimum of eight required to be included in main analyses). After all exclusions, 13 labs were included in the pre-registered analyses, which included a total of 152 participants (see Table 1 for Descriptives).

### 4.2 Stimuli

**Figure 1.**
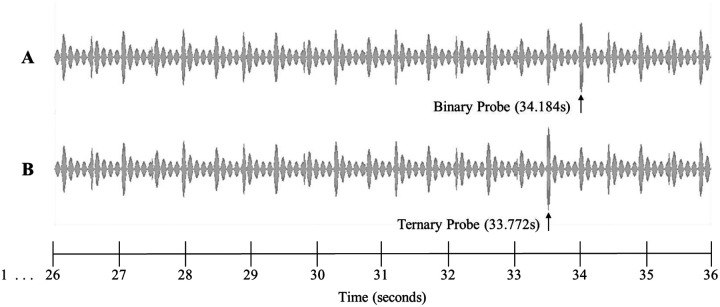
10 s excerpts of the 36 s auditory stimulus (x axis: time, y axis: sound amplitude). Note that this stimulus was extended by 3 s from the original stimulus used by Nozaradan et al. (2011), in order to allow imagery to be maintained for the same amount of time as the original study before introducing the probe tones. The probe tone (880 Hz) was superimposed onto the stimulus for 40ms. A) Binary probe tone, superimposed starting at 34.184 s. B) Ternary probe tone, superimposed starting at 33.772 s.

The stimulus, which was 33 s in duration, was obtained directly from the first author of Nozaradan et al. (2011). It consisted of a 333.3 Hz pure tone, which was amplitude-modulated with a 2.4 Hz periodicity, using an asymmetrical Hanning envelope (22 ms rise time and 394 ms fall time, amplitude modulation between 0 and 1) and then amplitude-modulated using an 11 Hz sinusoidal function, generating small irregularities resulting in a pseudo-periodic structure. To create a behavioral measure of conscious perception, we asked listeners to evaluate the fit of a probe that occurred after the initial 33 s stimulus. To do this, 3 s of the waveform were copied from the original and appended to the end of the stimulus and a probe was presented that occurred at a target or non-target position (relative to the imagery condition). The probe tone was added to the original signal and was presented at 880 Hz for 40 ms. The probe tone always occurred on either a binary beat (34.184 s after the beginning of the trial) or a ternary beat (33.732 s after the beginning of the trial) (See Figure 1). The auditory stimuli were presented binaurally through earphones or speakers at a comfortable hearing level (approximately 65 dB SPL). If speakers were used, the experimenter was required to listen to masking sounds during the test trials.

### 4.3 Task

Participants performed the same three conditions that were performed in the original study: a control condition; a binary imagery condition; and a ternary imagery condition. Each condition was presented as a separate block of trials: 12 trials (control condition) or 10 trials (imagery conditions) during which the auditory stimulus was presented after a 3 s silence. Stimulus presentation was self-paced. First, participants completed the block of control trials, during which they detected a very short (4 ms) sound interruption, which was inserted in two of the trials. The two trials containing a short interruption were excluded from analyses.

During the second and third blocks, participants completed the binary imagery condition and ternary imagery condition, counterbalanced for order. They imagined a binary beat structure or a ternary beat structure in the stimulus. Before participants completed each of the binary and ternary imagery conditions, they completed a standardized training procedure with an experimenter. All labs were required to send a video of a training session with one pilot participant for all three tasks, which was reviewed and approved by the first author prior to starting data collection (see OSF project page for example video made by first author). During training, participants were instructed to begin their imagery as soon as the stimulus began, making the first sound they hear a strong beat, and to maintain this imagery as consistently as possible throughout the entire trial. To help them understand exactly how to perform the imagery conditions, participants were asked to perform overt movements (hand tapping) and to count aloud, first with the help of the experimenter and then alone. Participants then completed a practice trial with just imagery, in which they practiced not moving or counting aloud while maintaining the imagery. Once they were comfortable with the imagery task, participants practiced maintaining the imagery and judging whether a probe tone occurring near the end of the stimulus was on the beat. All aforementioned training trials could be repeated until the participant felt comfortable to move on. Finally, participants completed two practice trials that included a response to the probe (*probe task*) and a rating to indicate their success at maintaining the imagery throughout the entire trial (*imagery success rating*). For the probe task, participants reported whether the probe tone occurred on a strong beat or a weak beat. For the imagery success task, they indicated how well they maintained the imagery throughout the entire trial using a 7-point rating scale, in which 1 was “Completely Got Lost” and 7 was “Maintained the Entire Time”^5^. Participants had to respond correctly on a group of two probe trials (one “correct”, one “incorrect”) during training prior to advancing to the test trials. If the participant did not get both training trials correct, the trials were re-administered to the participant again in a randomized order. If the participant did not respond correctly to both training trials after four repetitions (a total of eight practice trials), the experiment moved on to the test trials. However, this participant was then excluded from the main analyses. Each participant completed 10 test trials during each imagery condition. Participating labs were provided with a detailed script for training. Finally, participants filled out a demographic questionnaire, including questions regarding music and dance experience. The experiment, not including EEG set-up time, took approximately 30 to 45 minutes, depending on time required to complete training with each participant.

### 4.4 Experiment Script

Participating laboratories were provided with a Presentation (Neurobehavioral Systems, Inc.) script that ran the study and collected all behavioral responses. In the case that a laboratory did not have a Presentation software license, they were instructed to use the RR project’s specific instruction manual on how to set the experiment up with their own software, and they were required to upload their script to the OSF website. If necessary, labs were permitted to adapt the experiment instructions to a language other than English. These labs were required to upload the translated materials as well as a translator statement. See Table S1 for specific details for each lab.

### 4.5 EEG Recording

Each lab used their own EEG systems to collect data while participants completed the three conditions described above. While participants were requested to refrain from movement during the experiment, it was still possible that participants would engage in small micro-movements while listening to the rhythmic auditory stimuli. Thus, participating labs had the option to contribute additional data by measuring overt rhythmic movements by placing two external electrodes, one over the Sternocleidomastoid Muscle (SCM) of the neck and one on the First Dorsal Interosseous (FDI) muscle of the dominant hand. Recording from the neck and hand was not required for participation, in case labs were unable to record movement activity but still wished to participate. Details about each lab’s EEG equipment, number of electrodes, and electrode placement are detailed in the Individual Lab Details (see Table S1). Following suggestions from the authors of the original study, we provided careful instruction and motivation to complete the task prior to each test block. Experimenters were asked to monitor compliance with the instructions and help motivate the participant to perform the imagery conditions to the best of their ability, either by staying in the room with the participant during the test trials or by monitoring a live video feed (see Appendix A for more details), and they recorded any observable movement in the session notes.

## 5 Data Analysis

The data analyses were conducted by the first author at University of Western Ontario in accordance with the preregistered analysis plan, available at the project OSF Website (https://osf.io/4xqwu/). All participating labs sent their raw EEG data with trial event codes indicating the beginning of trial times in each of the experimental blocks in one of the approved formats^6^. Participating labs were required to send a detailed description of how they generated their event code list, including how they accounted for latency issues (if any). Labs were also required to send their log of participants’ movement, behavioral data, and survey data in an excel workbook (see individual lab data summaries on OSF page). The core meta-analyses were based on amplitudes at the imagery- related frequencies (binary: 1.2 Hz, ternary: 0.8 Hz) for the three conditions (control task, binary imagery, ternary imagery). This RR reports the average amplitudes, behavioral responses, and effect sizes for each lab. The official EEG data processing steps were written without viewing the actual data and have been made publicly available.

### 5.1 Stopping Criteria and Exclusions

As part of registering to participate in this RR and prior to beginning data collection, each lab indicated whether it aimed to stop data collection after collecting a full sample of 16 participants or a half sample of eight participants. The stopping criteria were designed to ensure that each lab would meet the minimum data collection requirements for the protocol and that the decision to end data collection would not be influenced by the results of the study.

Data were pre-registered to be excluded if a participant did not fit the specified recruitment criteria, did not follow instructions, did not complete the experiment, or if the experiment was administered incorrectly for any reason (e.g., incorrect training procedures). Labs were asked to note this explicitly at the time of the experiment in a lab testing log before examining the data for that participant. Participants were also excluded if they were observed moving during EEG data collection during the auditory stimulus in any way that may have affected imagery (i.e., moving rhythmically, tapping their finger, hands, or feet, bobbing their head). Experimenters were asked to make note of whether each participant was observed moving in a periodic manner. Fifty-nine of the 212 total participants were excluded: 42 for failing imagery training, eight due to excessive EEG artifacts (present in > 50% of EEG data), four due to experimenter or equipment error, one for observed rhythmic movement, and four due to the lab having too few participants after exclusions (Lab 14).

### 5.2 EEG Analysis

All EEG analyses were performed identically to the original paper, using MATLAB (The MathWorks), EEGLAB toolbox (Delorme & Makeig, 2004), and FieldTrip (Oostenveld et al., 2010). Prior to collecting the full dataset, all labs were required to conduct the full study with one pilot participant and send the data to the parent lab. These data files were used to create the final data processing scripts, which followed the analysis plan posted to the project OSF page during pre-registration.

All participating labs were asked to record the EEG signals with a low-pass filter of 500 Hz and a sampling rate of at least 1000 Hz^7^. Actual sampling rates for each lab are included in Table S1. In order to minimize the amount of resampling conducted on the data, labs reported their EEG system requirements and limitations prior to data collection, and the default sampling frequency [1000 Hz or 1024 Hz] was set to the sampling frequency most common among the EEG systems being utilized as of the data collection start date. Where necessary, data were downsampled to 1000 Hz or 1024 Hz (see Table S1 for individual lab details). Prior to completing any further processing steps, the first author inspected the raw data for all subjects to ensure no excessive noise or artifacts were present. At this point, eight participants were excluded due to an excessive percentage of data including artifacts (> 50% of the data). Data were referenced to the average of all EEG electrodes and filtered using a 0.1 Hz high-pass Butterworth zero-phase filter to remove very slow drift in the recorded signals. EEG epochs began 1 s after the onset of the stimulus and lasted 32 s. Artifacts produced by eye blinks/saccades and muscles were removed using independent component analysis (ICA) (Jung et al., 2000), using the runica algorithm (Makeig et al., 1996). Selections of ICA components for removal were conducted by two independent researchers (K. Nave and T. Chabin) and compared for reliability (see the pre-registered analysis plan on the OSF page for more details). During ICA manual selection, researchers were blind to participant and condition. After ICA rejection, for each subject and condition, averages were conducted across trials and transformed using a discrete Fourier transform (Frigo & Johnson, 1998) with a frequency resolution of 0.03 Hz, producing a frequency spectrum ranging from 0 to 500 Hz. The contribution of residual noise was removed by subtracting the average amplitude measured at neighboring frequency bins (two frequency bins ranging −0.15 to −0.09 Hz and from +0.09 to +0.15 Hz relative to each bin) at each bin of the frequency spectra. Stimulus- and imagery- related frequency responses were estimated by averaging the signal amplitude measured at the three frequency bins centered on the target frequency. The magnitude of these responses was averaged for each participant, condition, and target frequency across all scalp electrodes. Recordings from overt movements made by the neck and hand are reported and analyzed in the Exploratory Analysis section.

### 5.3 One-Way ANOVAs and Post-Hoc Tests on Effect of Condition

As in the original study, we conducted repeated-measures one-way ANOVAs to test the effect of condition (control, binary imagery, ternary imagery) on the amplitude of brain activity at the stimulus frequency (2.4 Hz), each of the imagery-related frequencies (binary: 1.2 Hz, ternary: 0.8 Hz), and the first upper harmonic of the ternary frequency (1.6 Hz), separately for each lab and across the entire collected sample. To compare the effect of condition for the four frequencies of interest (ternary (0.8Hz), binary (1.2Hz), ternary harmonic (1.6Hz), and stimulus (2.4Hz)) with the original study, the results with estimated effect sizes are provided for each participating lab, across all labs combined, and for Nozaradan et al. (2011) in Table 2.

The original paper also reported post-hoc tests, which revealed that both imagery-related frequencies only demonstrated higher amplitudes of the brain response when the imagery frequency matched the imagery performed (e.g., the binary frequency was enhanced when participants performed the binary imagery, but not when they performed the ternary imagery or during the control condition). To compare the effect of condition for the three frequencies of interest (binary, ternary, and stimulus) with the original study, these are provided for each participating lab in Table 3.

### 5.4 Meta-Analytic Estimates on the Effect of Imagery

The intended analyses were pre-registered and tested on simulated data before inspecting the data. The RR measured the meta-analytic across-lab effects of the raw mean differences between conditions in frequency-domain amplitudes of the brain responses, where the conditions compared were: the Control (C) task (e.g., in which no imagery took place), the Binary Imagery (B) task (e.g., in which beats were imagined on every other stimulus event), and the Ternary Imagery (T) task (e.g., in which beats were imagined on every third stimulus event). The original 2011 paper reported a statistically significant effect of condition (3-way Univariate ANOVA) on the amplitude at imagery frequencies based on the beat pattern that the participant was asked to imagine, as well as post hoc significant comparisons between the conditions as expected (0.8 Hz: T > C, T > B; 1.2Hz: B > C, B > T). In order to examine these condition comparisons, we used random effects meta-analyses to estimate four raw effect sizes, calculated as the difference in mean amplitude of brain activity between two conditions at each of the two beat imagery frequencies (i.e., ternary: 0.8 Hz, binary: 1.2 Hz): 1) Binary Control Effect: binary imagery minus control task at the binary frequency (1.2Hz), 2) Binary Active Effect: binary imagery minus ternary imagery at the binary frequency (1.2Hz), 3) Ternary Control Effect: ternary imagery minus control task at the ternary frequency (0.8Hz), and 4) Ternary Active Effect: ternary imagery minus binary imagery at the ternary frequency (0.8Hz). In all four cases, a significant positive estimate would provide evidence for the conclusions of the original study, suggesting a positive modulation of the SSEP amplitude for the imagery-related frequency. In addition, the RR implemented mixed-effects meta-analyses to test music experience and dance experience as moderating variables. The meta-analytic estimates are provided both with and without the moderators included. These random- and mixed-effects meta-analyses were conducted using the R package metaphor (Viechtbauer, 2010).

### 5.5 Logistic Regression to Predict Beat-Related Task Performance

If the effects observed in the original study indeed reflect the imagined beat pattern, then it would be expected that accuracy on the probe task would be related to the amplitude of brain activity at imagined beat frequencies. In addition, prior work suggests that musicians and dancers may have enhanced sensitivity to rhythmic organization (Palmer & Krumhansl, 1990), as well as enhanced beat-related brain responses (Jongsma et al., 2004). To test the contribution of these factors to the size of the beat-related brain response, we conducted a logistic regression to predict performance on the probe task on a trial-by-trial basis. Factors entered in the regression included participant ID, lab ID, music experience, dance experience, imagery type, probe type (ON beat vs. OFF beat), EEG amplitude at the beat imagery frequency, and EEG amplitude at the stimulus frequency.

## 6 Results

The goals of this RR were to 1) provide a precise measure of the size of the effects of beat imagery, and 2) extend the findings by relating the magnitude of the effects to a) a behavioral measure of beat perception and b) music/dance training of the participants – by combining the results from multiple, independently conducted studies. The results of all contributed studies are included in the analyses reported here regardless of their outcome, providing an unbiased meta-analysis of the effects. The analysis does not focus on null-hypothesis significance testing, but rather on the meta-analytic effect size for each outcome, with the confidence intervals estimated.

### 6.1 Descriptive Statistics

**Figure 2.**
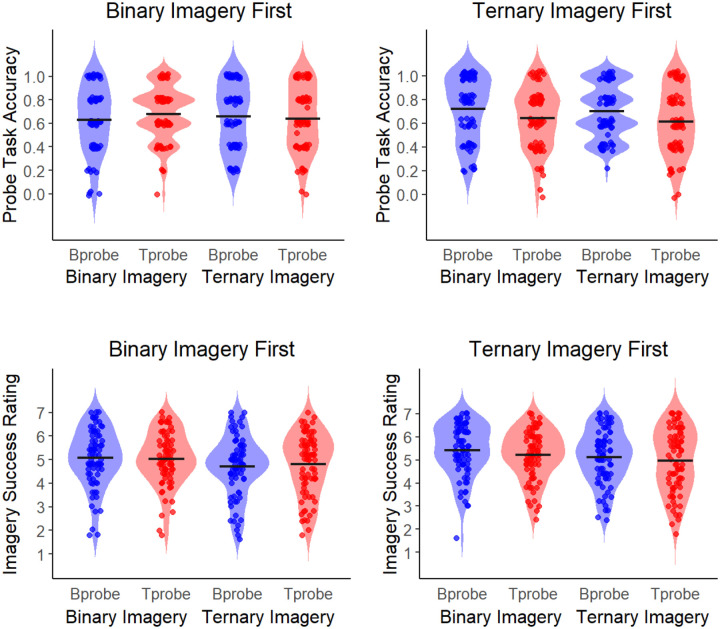
Average probe task accuracy (**top**) and average imagery success rating (**bottom**) across subjects from all participating labs on the behavioral task during imagery conditions. Performance on the binary probe trials is shown in blue and performance on the ternary probe trials is shown in red. Each dot represents an individual participant (*N* = 152) and the line represents the mean.

**Figure 3.**
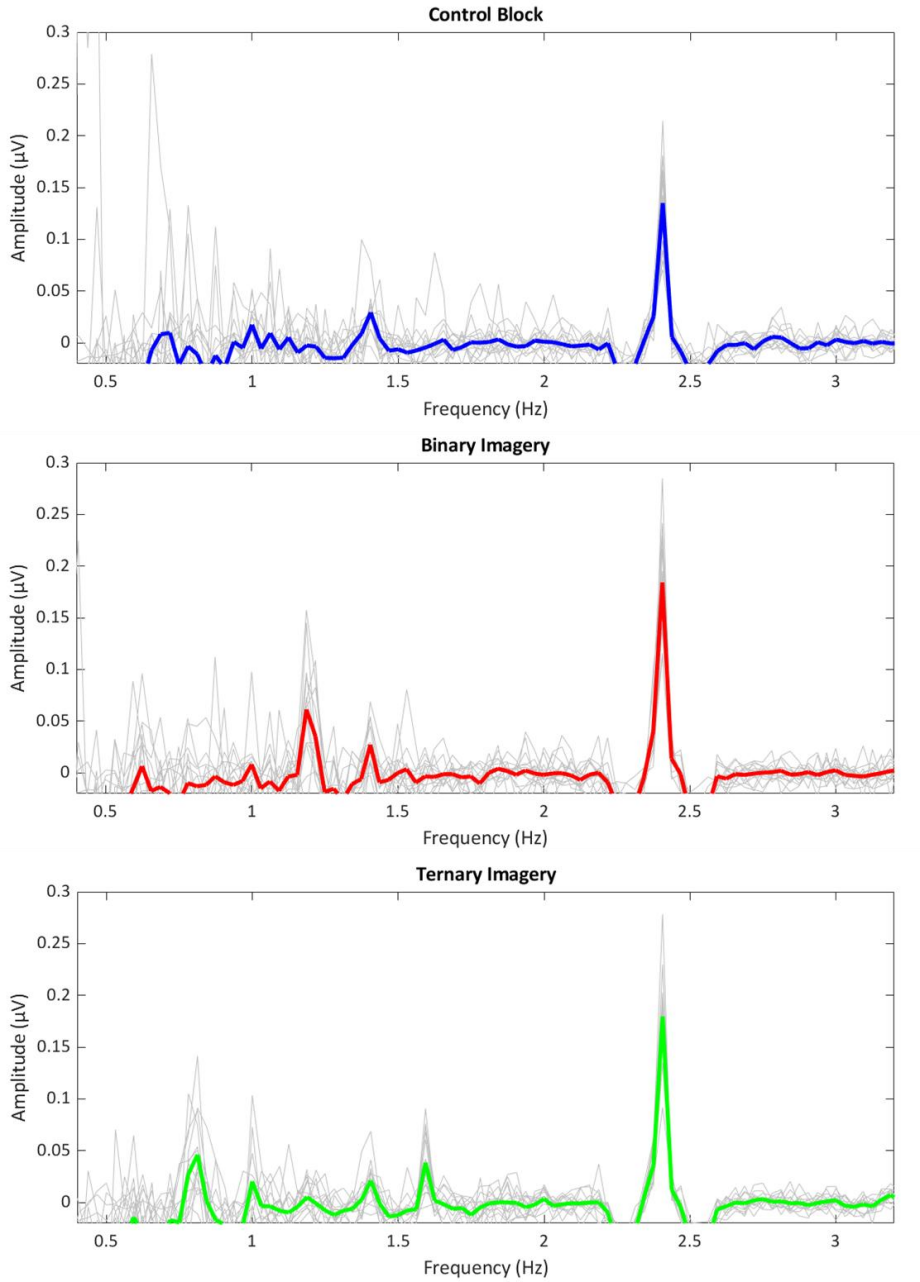
Lab-level average of the steady state-evoked potentials (SSEPs) elicited by the 2.4 Hz auditory stimulus in the control condition (top, blue), the 2.4 Hz stimulus plus binary imagery condition (middle, red), and the 2.4 Hz stimulus plus ternary imagery condition (bottom, green).The lab-level average frequency spectra are shown using a thick colored line, while single-lab averaged spectra are shown in thin gray lines. The frequency spectra represent the amplitude of the EEG signal (in microvolts) as a function of frequency, averaged across all scalp electrodes, after applying the noise subtraction procedure.

Descriptive statistics for each contributed study are provided in Table 1. Probe task accuracy was computed as the average correct responses across test trials, where a binary probe tone was indicated as “ON beat” during binary imagery only, and a ternary probe tone was indicated as “ON beat” during ternary imagery. Imagery success rating was computed as the average imagery success rating given (1 = “Completely got lost”, 7 = “maintained imagery the entire time”) across test trials. In addition, the average amplitude of brain activity at the stimulus frequency, binary frequency, and ternary frequency is provided for each condition, along with average performance on all behavioral tasks. Results from the probe task measures are presented in Figure 2. Average frequency spectra for each lab and across all labs are displayed in Figure 3.

#### 6.2.1 Effect of Condition on Brain Activity at Binary Frequency (1.2 Hz)

##### Binary Control Effect.

**Figure 4.**
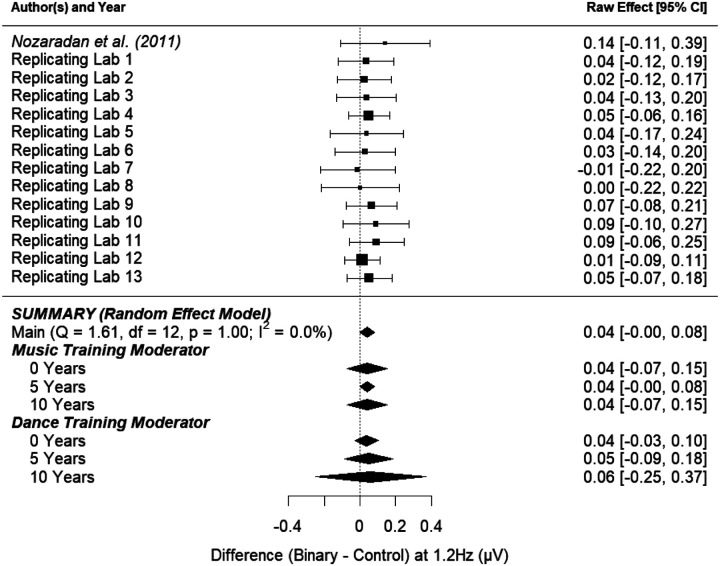
Point-estimate for the meta-analytic effect of condition (control task vs. binary imagery) on the binary frequency. Studies are listed alphabetically by the last name of the first author for that lab’s contribution, with the original study presented at the top. Squares indicate each lab’s mean difference, where the size of the square corresponds to the inverse of the standard error of the difference score, and the error bars indicate 95% confidence intervals (CI) around the mean difference. Diamonds indicate the random-effects meta-analytic effect size estimate, where the width represents the 95% CI. The first diamond represents a meta-analysis with no moderators. The next three diamonds represent the meta-analytic estimate with music training included as a moderator, and the final three diamonds represent the meta-analytic estimate with dance training included as a moderator. Note that none of the meta-analyses include the original Nozaradan et al. (2011) result.

Figure 4 shows the point-estimate for the meta-analytic effect of condition on the binary frequency (1.2 Hz), in which the raw effect corresponds to the difference between the mean amplitudes in the control task and the binary imagery condition. Our meta-analysis revealed an average raw mean difference of 0.041, such that the amplitude of brain activity was 0.041 μV higher during binary imagery compared to the control task. This effect ranged from −0.012 to 0.093 across the included studies. The 95% meta-analytic confidence interval ranged from −0.0004 to 0.082, overlapping with zero (*p* = .052). In none of the 13 replication attempts did the 95% confidence interval overlap with the raw effect size from Nozaradan et al. (2011) (0.12); all 13 intervals were smaller than the one reported in Nozaradan et al. (2011).

The variability in the effect size among the studies (i.e., heterogeneity) was consistent with what would be expected by chance (*τ* = 0.00, *I*^*2*^ = 0.00%, *H*^*2*^ = 1.00, *Q*_*13*_ = 1.606, *p* = .999). The observed homogeneity among the study effect sizes suggests that moderating variables may have little to no effect on the meta-analytic estimate of the effect. Figure 4 shows that the effect of condition (control task vs. binary imagery) on the amplitude of brain activity at the binary frequency was not substantially moderated by music training and was not substantially moderated by dance training.

##### Binary Active Effect.

**Figure 5.**
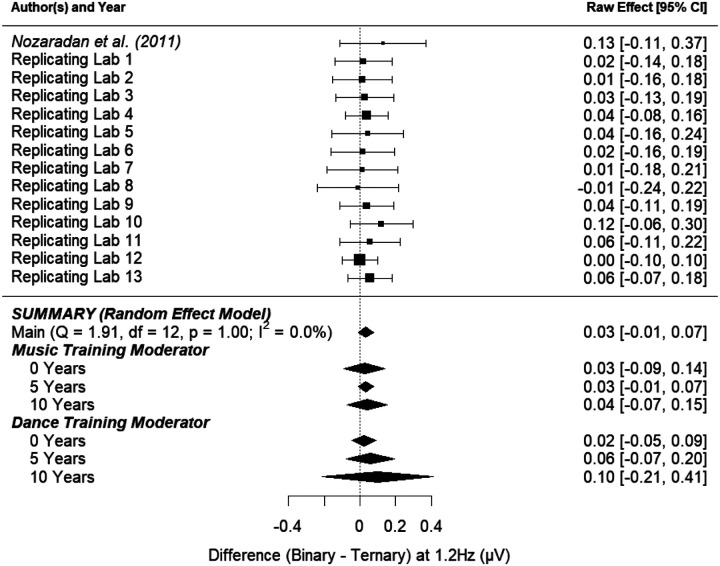
Point-estimate for the meta-analytic effect of condition (binary imagery vs. ternary imagery) on the binary frequency. Studies are listed alphabetically by the last name of the first author for that lab’s contribution, with the original study presented at the top. Squares indicate each lab’s mean difference, with the size of the square corresponding to the inverse of the standard error of the difference score, and the error bars indicate 95% confidence intervals (CI) around the mean difference. Diamonds indicate the random-effects meta-analytic effect size estimate, where the width represents the 95% CI. The first diamond represents a meta-analysis with no moderators. The next three diamonds represent the meta-analytic estimate with music training included as a moderator, and the final three diamonds represent the meta-analytic estimate with dance training included as a moderator. Note that none of the meta-analyses include the original Nozaradan et al. (2011) result.

Figure 5 shows the point-estimate for the meta-analytic effect of condition on the binary frequency, in which the raw effect corresponds to the difference between the binary imagery condition and the ternary imagery condition. Our meta-analysis revealed an average raw mean difference of 0.032 μV, such that the amplitude of brain activity was 0.032 μV higher during binary imagery compared to ternary imagery. This effect ranged from −0.012 to 0.120 across the included studies. The 95% meta-analytic confidence interval ranged from −0.010 to 0.075, overlapping with zero (*p* = .132). In one out of 13 replication attempts, the 95% confidence interval overlapped the raw effect size from Nozaradan et al. (2011) (0.12); of the 12 intervals that did not overlap the original effect size, all were smaller than the one reported in Nozaradan et al. (2011).

The variability in the effect size among the studies (i.e., heterogeneity) was consistent with what would be expected by chance (*τ* = 0.00, *I*^*2*^ = 0.00%, *H*^*2*^ = 1.00, *Q*_*13*_=1.907, *p* = .999). The observed homogeneity among the study effect sizes suggests that moderating variables may have little to no effect on the meta-analytic estimate of the effect. Figure 5 shows that the overall effect of condition (binary imagery vs. ternary imagery) on the amplitude of brain activity at the binary frequency was not substantially moderated by music training and was not substantially moderated by dance training.

#### 6.2.2 Effect of Condition on Brain Activity at Ternary Frequency.

##### Ternary Control Effect.

**Figure 6.**
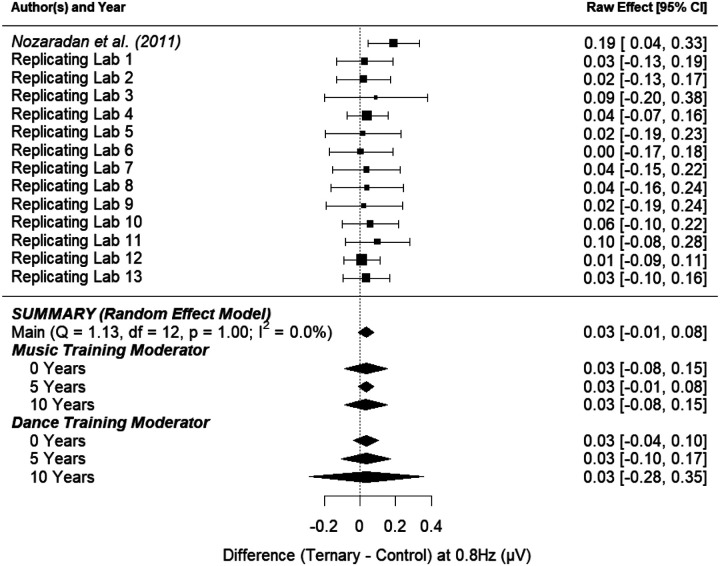
Point-estimate for the meta-analytic effect of condition (control task vs. ternary imagery) on the ternary frequency. Studies are listed alphabetically by the last name of the first author for that lab’s contribution, with the original study presented at the top. Squares indicate each lab’s mean difference, where the size of the square corresponds to the inverse of the standard error of the difference score, and the error bars indicate 95% confidence intervals (CI) around the mean difference. Diamonds indicate the random-effects meta-analytic effect size estimate, where the width represents the 95% CI. The first diamond represents a meta-analysis with no moderators. The next three diamonds represent the meta-analytic estimate with music training included as a moderator, and the final three diamonds represent the meta-analytic estimate with dance training included as a moderator. Note that none of the meta-analyses include the original Nozaradan et al. (2011) result.

Figure 6 shows the point-estimate for the meta-analytic effect of condition on the ternary frequency, where the raw effect corresponds to the difference between the control task and the ternary imagery condition. Our meta-analysis revealed an average raw mean difference of 0.034, such that the amplitude of brain activity was 0.034 μV higher during ternary imagery compared to the control task. This effect ranged from 0.005 to .149 across the included studies. The 95% meta-analytic confidence interval ranged from −0.010 to 0.077, overlapping with zero (*p* = .126). In three out of 13 replication attempts, the 95% confidence interval overlapped the mean effect size from Nozaradan et al. (2011) (0.12); of the 10 intervals that did not overlap the original effect size, all were smaller than the one reported in Nozaradan et al. (2011).

The variability in the effect size among the studies (i.e., heterogeneity) was consistent with what would be expected by chance (*τ* = 0.00, *I*^*2*^ = 0.00%, *H*^*2*^ = 1.00, *Q*_*13*_ = 1.132, *p* = 1.00). The lack of unexplained variability among the study effect sizes suggests that moderating variables may have little to no effect on the meta-analytic estimate of the effect. Figure 6 shows that the overall effect of condition (control task vs. ternary imagery) on the amplitude of brain activity at the ternary frequency was not substantially moderated by music training and was not substantially moderated by dance training.

##### Ternary Active Effect.

**Figure 7.**
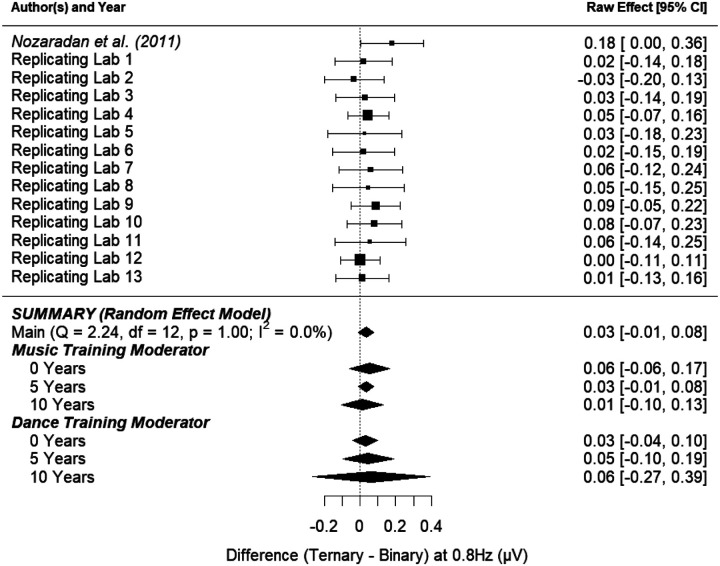
Point-estimate for the meta-analytic effect of condition (ternary imagery vs. binary imagery) on the ternary frequency. Studies are listed alphabetically by the last name of the first author for that lab’s contribution, with the original study presented at the top. Squares indicate each lab’s mean difference, where the size of the square corresponds to the inverse of the standard error of the difference score, and the error bars indicate 95% confidence intervals (CI) around the mean difference. Diamonds indicate the random-effects meta-analytic effect size estimate, where the width represents the 95% CI. The first diamond represents a meta-analysis with no moderators. The next three diamonds represent the meta-analytic estimate with music training included as a moderator, and the final three diamonds represent the meta-analytic estimate with dance training included as a moderator. Note that none of the meta-analyses include the original Nozaradan et al. (2011) result.

Figure 7 shows the point-estimate for the meta-analytic effect of condition on the ternary frequency, with the raw effect corresponding to the difference between the ternary imagery condition and the binary imagery condition. Our meta-analysis revealed an average raw mean difference of 0.035, such that the amplitude of brain activity was 0.035 μV higher during ternary imagery compared to binary imagery. This effect ranged from −0.033 to 0.089 across the included studies. The 95% meta-analytic confidence interval ranged from −0.008 to 0.077, overlapping with zero (*p* = .111). In three out of 13 replication attempts, the 95% confidence interval overlapped the raw effect size from Nozaradan et al. (2011) (0.12); of the 10 intervals that did not overlap the original effect size, all were smaller than the one reported in Nozaradan et al. (2011).

The variability in the effect size among the studies (i.e., heterogeneity) was consistent with what would be expected by chance (*τ* = 0.00, *I*^*2*^ = 0.00%, *H*^*2*^ = 1.00, *Q*_*13*_ = 2.237, *p* = .999). The observed homogeneity among the study effect sizes suggests that moderating variables may have little to no effect on the meta-analytic estimate of the effect. Figure 7 shows that the overall effect of condition (ternary imagery vs. binary imagery) on the amplitude of brain activity at the ternary frequency was not substantially moderated by music training, and was not substantially moderated by dance training

#### 6.2.3 Summary of Meta-Analytic Effects of Condition

The meta-analytic estimates of the effect of condition on the imagery-related SSEP amplitude estimate a raw difference of .03 μV– .04 μV when comparing the means of the corresponding beat imagery condition to either the other imagery condition or the control condition. All four estimated effect sizes had confidence intervals overlapping zero, suggesting the effect sizes are not significantly above chance (*p* ranges from p = .052 to p = .132). However, it is important to note that of the thirteen labs included in this analysis, eight produced data that violated assumptions of sphericity and thus required a Greenhouse Geiser correction (see Table 1). Thus, we also conducted our pre-registered meta-analytic estimates using raw differences between medians, instead of means, for the four estimated effect sizes in an exploratory analysis (see section 7.2).

### 6.3 One-Way ANOVAs and Post-Hoc Tests on Effect of Condition

Table 2 shows the resulting effect sizes (as measured by partial eta squared) of repeated measures one-way ANOVAs conducted for each individual lab on the effect of condition (control, binary, ternary), in which the dependent variable is the amplitude of brain activity at the frequency of interest. The results of the original study are shown at the top, followed by all participating labs. Table 3 shows the results of post hoc t-tests conducted for each individual lab following the one-way ANOVAs for each frequency of interest. Specifically, these post hoc tests are conducted in accordance with the original study, such that for each lab, a t-test is conducted to demonstrate any significant difference in amplitude of the brain activity between any two conditions (control vs. binary imagery, control vs. ternary imagery, and binary imagery vs. ternary imagery) at each of the frequencies of interest (stimulus [2.4 Hz], binary [1.2 Hz], ternary [0.8 Hz], and 1^st^ ternary harmonic [1.6 Hz]), resulting in nine *t*-tests for each of the participating labs. Results are reported for each lab, including the original study.

In addition, correlations were conducted to investigate the relation between the magnitude of the brain activity at the imagery-related frequency, music training (in years), dance training (in years), and ratings of imagery success and probe task accuracy. These are presented below in Table 4, both for each individual lab and as a grand average.

### 6.4 Relation Between Brain Activity and Other Factors

**Figure 8.**
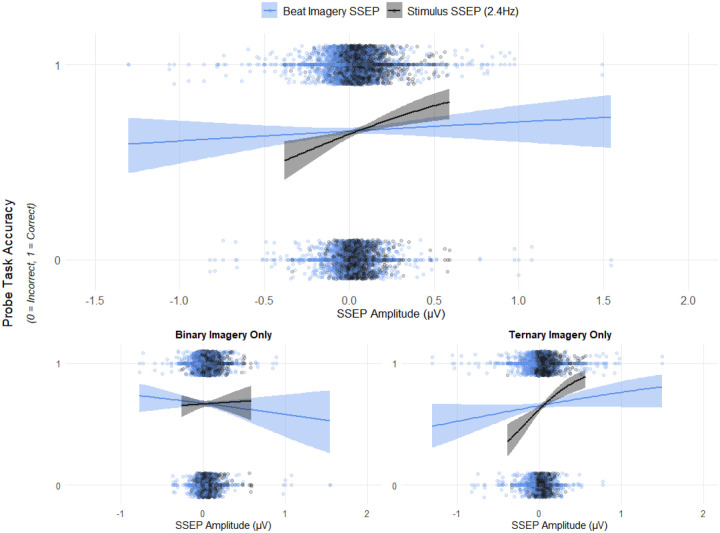
Top: Results of trial-by-trial logistic regression examining whether frequency-tagged neural responses predict imagery-related task performance. Two predictors of accuracy, [blue: amplitude at the beat imagery frequency; black: amplitude at the stimulus frequency], are plotted along the x-axis as the amplitude (in microvolts) of the EEG signal. Trial-by-trial accuracy on the probe task is plotted on the y-axis (0: incorrect response, 1: correct response). Bottom: Data plotted separately by imagery type.

Factors entered into the regression included participant ID, lab ID, music experience, dance experience, imagery type, trial type (ON beat vs OFF beat), EEG amplitude at the imagined beat frequency, and EEG amplitude at the stimulus frequency. Results showed that stimulus-frequency amplitude (Wald χ^2^ = 4.944, *p* = .026) was a significant predictor of trial accuracy, such that a higher magnitude of brain activity at the stimulus-frequency predicted higher accuracy on the probe task. No other significant predictors were observed. The results of this logistic regression are plotted in Figure 8.

## 7 Exploratory Analyses

### 7.1 Effect of Micro-Movements on SSEPs to Imagined Beat

While participants were asked to refrain from movement during the three conditions and the experimenter recorded any observable rhythmic movement by the participant (only observed in 1 participant, who was excluded), it is still possible that participants performed unobservable micro-movements to maintain the rhythmic structure, and such movement could contaminate the EEG response. Because the procedure to measure muscle activity was optional (SCM muscle in the neck and the FDI muscle in the hand) and due to the additional constraints of the COVID-19 pandemic, only one lab was able to collect such data (Lab #8). Of the eight participants from this lab (after pre-registered exclusions), only four participants had viable data from the hand and neck (due to issues in the signal reported by the experimenter). Thus, we did not have sufficient power to estimate the contribution of overt movement to the measured imagery -related SSEPs. Despite this, the movement log recorded by experimenters indicated that only one participant was observed moving rhythmically and was excluded. In addition, more recent research suggests that unobservable micromovements are not likely to contribute to the imagery-related SSEPs during rhythmic imagery, confirmed via both EMG response and motion capture (Cheng et al., 2021).

### 7.2 Meta-Analyses Using Median Raw Differences

After conducting the Univariate ANOVAs for the individual labs (see section 6.4 in Results and Table 2), it was clear that several lab samples violated the assumption of sphericity. Similar to the original study, which reports medians for each condition instead of means, we decided to also examine the four meta-analytic effects on raw differences between medians. Exploratory meta-analyses on medians were conducted using the R package metamedian (McGrath et al., 2024).

#### 7.2.1 Effect of Condition on Brain Activity at Binary Frequency.

##### Binary Control Effect.

Figure S2 shows the point-estimate for the meta-analytic effect of condition on the binary frequency, in which the raw effect corresponds to the median difference between the control task and the binary imagery condition. Our meta-analysis revealed an average raw median difference of 0.025, such that the amplitude of brain activity was 0.025 μV higher during binary imagery compared to the control task. This effect ranged from −0.002 to 0.092 across the included studies. The 95% meta-analytic confidence interval ranged from 0.014 to 0.049, not overlapping with zero (*p* < .001). In 0 out of 13 replication attempts, the third interquartile range overlapped the effect size from Nozaradan et al. (2011) (.12); 11 of the 13 intervals that did not overlap the original effect size were smaller than the one reported in Nozaradan et al. (2011). The variability in the effect size among the studies (i.e., heterogeneity) was larger than what would be expected by chance (*τ* = 0.0002, *I*^*2*^ = 49.6%, *H*^*2*^ = 1.99, *Q*_*13*_ = 25.901, *p* = .011). The significant variability among the study effect sizes leaves open the possibility that the strength of the effect varies as a function of one or more moderating variables.

##### Binary Active Effect.

Figure S3 shows the point-estimate for the meta-analytic effect of condition on the binary frequency, in which the raw effect corresponds to the median difference between the binary imagery condition and the ternary imagery condition. Our meta-analysis revealed an average raw median difference of 0.024, such that the amplitude of brain activity was 0.024 μV higher during binary imagery compared to ternary imagery. This effect ranged from −0.010 to 0.104 across the included studies. The 95% meta-analytic confidence interval ranged from 0.013 to 0.036, not overlapping with zero (*p* < .001). In none out of 13 replication attempts did the third interquartile range overlapped the effect size from Nozaradan et al. (2011) (0.12); Of the 13 intervals that did not overlap the original effect size, all were smaller than the one reported in Nozaradan et al. (2011). The variability in the effect size among the studies (i.e., heterogeneity) was larger than what would be expected by chance (*τ* = 0.0003, *I*^*2*^= 60.5%, *H*^*2*^ = 2.53, *Q*_*13*_ = 30.047, *p* = .003). The significant variability among the study effect sizes leaves open the possibility that the strength of the effect varies as a function of one or more moderating variables.

#### 7.2.2 Effect of Condition on Brain Activity at Ternary Frequency.

##### Ternary Control Effect.

Figure S4 shows the point-estimate for the meta-analytic effect of condition on the ternary frequency, where the raw effect corresponds to the difference between the control task and the ternary imagery condition. Our meta-analysis revealed an average raw median difference of 0.029, such that the amplitude of brain activity was 0.029 μV higher during ternary imagery compared to the control task. This effect ranged from 0.001 to 0.072 across the included studies. The 95% meta-analytic confidence interval ranged from 0.018 to 0.039, not overlapping with zero (*p* < .001). In 2 out of 13 replication attempts, the third interquartile range overlapped the effect size from Nozaradan et al. (2011) (0.12); Of the 11 intervals that did not overlap the original effect size, all were smaller than the one reported in Nozaradan et al. (2011). The variability in the effect size among the studies (i.e., heterogeneity) was consistent with what would be expected by chance (*τ* = 0.0001, *I*^*2*^ = 29.3%, *H*^*2*^ = 1.42, *Q*_*13*_ = 15.571, *p* = .212). The observed homogeneity among the study effect sizes suggests that moderating variables may have little to no effect on the meta-analytic estimate of the effect.

##### Ternary Active Effect.

Figure S5 shows the point-estimate for the meta-analytic effect of condition on the ternary frequency, with the raw effect corresponding to the difference between the ternary imagery condition and the binary imagery condition. Our meta-analysis revealed an average raw median difference of 0.031, such that the amplitude of brain activity was 0.031 μV higher during ternary imagery compared to binary imagery. This effect ranged from −0.02 to .097 across the included studies. The 95% meta-analytic confidence interval ranged from 0.015 to 0.049, not overlapping with zero (*p* < .001). In 3 out of 13 replication attempts, the third interquartile range overlapped the effect size from Nozaradan et al. (2011) (0.12); Of the 10 intervals that did not overlap the original effect size, all were smaller than the one reported in Nozaradan et al. (2011). The variability in the effect size among the studies (i.e., heterogeneity) was larger than what would be expected by chance (*τ* = 0.0006, *I*^*2*^ = 0.714%, *H*^*2*^ = 3.49, *Q*_*13*_ = 39.586, *p* < .0001). The significant variability among the study effect sizes leaves open the possibility that the strength of the effect varies as a function of one or more moderating variables.

#### 7.2.3 Summary of Exploratory Meta-Analytic Effects of Condition

Given the violations of sphericity present in the individual lab’s data, the use of medians is more appropriate to examine differences among conditions, compared to the pre-registered estimates of differences among means (see section 6.3). The exploratory meta-analytic estimates of the effect of condition on the imagery-related SSEP amplitude estimate a raw difference of .02 μV– .03 μV when comparing the medians of the corresponding beat imagery condition to either the other imagery condition or the control condition. All four of the exploratory estimated effect sizes had confidence intervals that did not overlap zero, suggesting the effects due to experimental condition are significantly above chance (all *p*’s < .001). Despite the resulting evidence for significant effects related to the experimental manipulation (i.e., beat imagery), the size of the estimated effects is indeed quite small. Furthermore, while all four of the preregistered meta-analytic estimates (using differences of means) suggest homogeneity in data, three of the four exploratory meta-analytic estimates using medians suggest the data are heterogeneous, leaving open the possibility that other moderating variables may explain variability among the contributing labs. Unfortunately, the R package used for meta-analytic estimates of medians (used for the current exploratory analysis) does not offer the capability to test for moderating variables. Future research should focus on potential moderating variables to these effects, including not only years of music training, but also more precise measures of individual differences in beat perception ability, such as by measuring musical sophistication (e.g., using the Goldsmiths Musical Sophistication Index, Müllensiefen et al., 2014)) or more specifically beat synchronization ability (e.g., using the Beat Alignment Test (BAT), Iversen & Patel, 2008).

### 7.3 Analysis of Behavioral Responses on Probe Task

To examine variability in performance on the Probe Task on the basis of our experimental factors, we conducted a mixed effects ANOVA for both probe task accuracy and imagery success ratings, each with two between subject factors (imagery order, lab membership) and two within subject factors (imagery type, probe type), where imagery order has two levels (binary first, ternary first), lab membership has thirteen levels, imagery type has two levels (binary, ternary), and probe type has two levels (“ON” beat, “OFF” beat).

Regarding probe task accuracy, there was a significant main effect of lab, *F*(1,126) = 2.067, *p* = .024, *η*_*p*_^*2*^= .16, and a significant probe type x imagery order interaction, *F*(1,126) = 7.808, *p* = .006, *η*_*p*_^*2*^= .06, with no other significant main effects nor interactions (all *p* > .05). Post-hoc tests revealed no significant differences among labs after correcting for multiple comparisons. In addition, accuracy was significantly greater for the binary probe than the ternary probe trials, but only for participants who completed ternary imagery first, *t*(126) = 3.321, *p* = .007, *Cohen’s d* = .30. Regarding imagery success ratings, there was a significant main effect of imagery type, *F*(1,126) = 15.899, *p* < .001, *η*_*p*_^*2*^= .11, and a significant probe type x imagery order interaction, *F*(1,126) = 6.205, *p* = .014, *η*_*p*_^*2*^= .05, with no other significant main effects or interactions (*p* > .05). Post hoc tests revealed significantly greater imagery success ratings for binary imagery compared to ternary imagery, *t*(126) = 3.987, *p* < .001, *Cohen’s d* = .26. Post-hoc comparisons exploring the probe type x imagery order interaction were all non-significant after correcting for multiple comparisons. Results from the probe task are presented in Figure 2. There was a significant correlation between probe task accuracy and imagery success ratings (*r* = 0.36, *p* < .001).

### 7.4 Relation Between Brain Activity and Other Factors by Imagery Condition

In order to further explore the relation between brain activity and behavioral performance, we performed the pre-registered Logistic Regression separately for each Imagery condition, removing “imagery type” as a factor. For Ternary imagery trials, stimulus-frequency amplitude (Wald χ^2^ = 13.235, *p* < .001) and probe type (Wald χ^2^ = 6.743, *p* = .009) were significant predictors of trial accuracy, such that a higher magnitude of brain activity at the stimulus-frequency predicted higher accuracy on the probe task, and more correct responses were given for the OFF probe than the ON probe. No other significant predictors were observed. For Binary imagery trials, none of the predictors were statistically significant.

## 8 General Discussion

This Registered Report included data from 13 laboratories for a total of 152 participants, after lab- and individual- dataset exclusions. The data were collected by following a vetted experimental design with a preregistered analysis plan. The first aim of this RR was to estimate the true effect sizes reported in the original 2011 study by performing meta-analyses across a multi-lab design. The overall results demonstrate that the original effect of imagery reported by Nozaradan and colleagues (imagining an amplitude modulated pure tone as having a binary or ternary stress pattern) is much smaller than originally reported when tested with a larger overall sample than the original study. In particular, the pre-registered meta-analytic effect sizes tested ranged from .03 to .04 Hz, whereas the original effect sizes ranged from .13 to .19 Hz, consistent with studies showing that replicated effects are typically smaller and less likely to be statistically significant than the original effects (Open Science Collaboration, 2015). While all four of the pre-registered meta-analytic effects (calculated with means for each lab) produced confidence intervals that overlapped with 0, exploratory analyses using medians (due to multiple violations of sphericity across labs) did result in all four meta-analytic effect sizes having confidence intervals that did not overlap with 0. Furthermore, the (non-pre-registered) analyses that included all 152 participants together did result in significant effects of imagining a beat on brain activity. This suggests that if the effect of periodic imagery on brain activity is a true effect, then sample sizes even larger than our overall sample are likely necessary to detect them reliably. Because the effect sizes related to condition comparisons (e.g., binary beat vs. ternary beat) are estimated by the current study to be quite small and require a very large sample to detect, frequency-tagging may not be a feasible method to explore neural correlates of musical beat perception. Importantly, while the repeated-measures ANOVA results of imagery produced significant effects of condition at the grand average level (*N* = 152) for the imagery-related frequencies (binary: 1.2 Hz, ternary: 0.8 Hz, 1.6 Hz), there was also an additional significant effect of condition at the stimulus frequency (2.4 Hz), such that imagery conditions resulted in larger SSEPs than the control condition. This may have implications for the effect of task demands on the size of SSEPs measured in response to auditory rhythm.

Due to the small sample size of the original study and the high amounts of musical training in those participants, we also tested for moderating effects of both musical and dance experience in our pre-registered meta-analyses. None of these moderation effects were significant. One weakness to this analysis is that only the average number of music years or dance years is considered for each lab, which reduces the influence of individual differences. To this point, we also conducted correlations between music/dance years of training and the four meta-analytic effects on a participant basis, and all correlations were minor and not significant (range of Pearson’s *r*: −.181 to .099, all *p*’s > .05). This finding is perhaps best considered inconclusive, however, because of the very small main effect of imagination on brain activity. Specifically, it is likely that any moderation by musical or dance experience would only change the imagination effect by a fraction of that effect; therefore, to reliably detect such a moderation effect would likely require several times the number of participants we tested across all the laboratories. Our data also showed no positive correlation between years of training and the amplitude of imagery-related SSEPs, even across our entire sample (N = 152). Contrary to our hypothesis, there was a significant negative correlation between music years of training and the amplitude at the ternary beat imagery across the sample. Probing such a link, assuming it exists, may need a more robust measure of musical aptitude and experience, in addition to years of training.

The second main aim of this RR was to assess the relationship between imagery-related neural responses (SSEP amplitude) and imagery-related behavioral responses. Surprisingly, the imagery-related stimulus SSEP amplitude (binary: 1.2 Hz, ternary: 0.8 Hz) was not related to the performance on the imagery-related task, but rather the amplitude of neural activity at the stimulus frequency (2.4Hz, the frequency of the periodic physical stimulus) was significantly predictive of performance, regardless of imagery condition. This raises questions about the assumption that the amplitude of the imagery-related SSEP responses reflects top-down perception of beat and meter. The data in the current study suggest that rather than representing the strength of beat or meter entrainment, SSEPs may reflect something else, such as how well a stimulus was encoded or how much attention the participant was directing to the task (Gibbings et al., 2023; Saupe et al., 2009). Few prior studies have related the modulation of beat-related SSEPs to behavioral responses from the listener. While some published work suggests correlations between SSEPs and groove ratings (Cameron et al., 2019; Zalta et al., 2024) and temporal perturbation/precision (Nozaradan et al., 2016), the extent to which perception-related SSEPs represent general timing mechanisms versus beat- or meter- related neural processes remains unclear.

Indeed, one explanation is that the current imagery task is not suitable to measure beat or meter perception. However, the current study demonstrates a similar pattern of results relating brain (SSEPs) to behavior (task-related listener responses) as a recent investigation of SSEPs to induced (and not imagined) musical beat (Nave et al., 2022). To our knowledge, this is the only other existing investigation of beat-related SSEPs that controlled for stimulus properties *and* statistically compared them to behavioral responses from a concurrent beat-related task. Listeners judged whether drum probes matched the beat of a rhythm after a musical context naturally induced one of two beat percepts in the rhythm. Crucially, while results suggested group-level differences in SSEP amplitudes as predicted between the two beat contexts, Nave and colleagues (2022) found evidence that beat-related SSEPs were not always positively related to performance on the beat-related task. The exploratory analyses regressing trial performance against the beat-related SSEP amplitude revealed a positive relation for some conditions (e.g., ternary beat at a faster tempo) – which mirrors the apparent positive relation between the ternary SSEP and performance on the ternary imagery task in the current study (see Figure 8), albeit not statistically significant – and a negative relation for others (e.g., binary beat as a slower tempo) – which mirrors the apparent negative relation between the binary SSEP and performance on the binary imagery task in the current study (see Figure 8), albeit not statistically significant. Thus, we suggest that frequency-tagged responses at the perceptual frequency (i.e., perceived beat or meter) do not necessarily correlate positively with listener performance on a perception-related task. It is important to consider stimulus properties in the analyses, and researchers should use caution when reverse-inferring that frequency-tagged beat-related responses indicate beat perception.

Even though the effect sizes we detected were much smaller than the original study, many studies have been published since Nozaradan et al. (2011) that examine effects on beat-related SSEPs. Some of these studies report positive evidence for non-stimulus-driven modulatory effects of attention and learning on SSEPs (Celma-Miralles & Toro, 2019; Chemin et al., 2014; Gibbings et al., 2023; Nave et al., 2022), similar to the Nozaradan et al. (2011) study. However, most studies demonstrate effects that are more likely to be stimulus driven, such as low-complexity/highly-periodic vs. high-complexity/weakly-periodic rhythms (Edalati et al., 2023; Lenc et al., 2023; Mathias et al., 2020; Nozaradan et al., 2016), music vs. speech (Harding et al., 2019; Vanden Bosch der Nederlanden et al., 2020), auditory vs. non-auditory modalities (Celma-Miralles et al., 2016; Gilmore & Russo, 2021), and rhythms composed of low- vs. high-frequency tones (Lenc et al., 2018). While beat-related SSEP amplitude differences have been observed in missing pulse rhythms (e.g., no energy at 2Hz in the stimulus), the estimated beat-related (1Hz) SSEPs were “averaged over 10% of the sensors with the highest 2 Hz amplitude”, thereby excluding 90% of their data where the missing frequency of 2Hz was not represented in the brain by the listener (Tal et al., 2017).

A positive feature of these SSEP studies since 2011 is that they generally used sample sizes larger than Nozaradan et al. (2011), which had a sample size of N = 8. Another positive feature of such studies is that they measure SSEPs (e.g., amplitude peaks in the spectrum of brain activity at frequencies of interest) that can be isolated at expected frequencies in amplitude spectra of EEG activity, and whose presence is clearly related to experimental stimulation or manipulation and not to other oscillatory activity such as ongoing brain rhythms. This assessment of generally strong frequency-domain peaks is based in part on visual inspection of their spectral data plots, although some studies statistically tested whether SSEP peaks were significantly above a background noise level. On the other hand, very few of them (e.g., Tierney & Kraus, 2015) used sample sizes close to our sample size, raising concerns that some if not most of these studies are reporting inflated estimates of the true population effects under investigation. Of particular concern is that some studies use sample sizes not much larger than Nozaradan et al. (2011) to compare multiple groups (e.g., Celma-Miralles & Toro, 2019; Cirelli et al., 2016; Laffere et al., 2021; Nozaradan et al., 2017; Stupacher et al., 2017) or to test correlations between SSEP amplitude and behavioral or individual difference measures (Cameron et al., 2019; Nguyen et al., 2023; Nozaradan et al., 2016; Tal et al., 2017; Zalta et al., 2024; Zhao & Kuhl, 2020), even though such effects typically require relatively large sample sizes (Brysbaert, 2019). Relatedly, frequency tagging approaches have become increasingly used by researchers to investigate entrainment to beat-related frequencies in preverbal infants, even though infants are likely to have far noisier data than adult participants (Edalati et al., 2023; Flaten et al., 2022; Lenc et al., 2023; Nguyen et al., 2023). Nevertheless, these studies do not typically test larger numbers of participants to compensate for the extra noise compared to adult studies, which themselves are likely to be underpowered.

This raises concerns of false positives in SSEP studies overall due to the file drawer effect or p-hacking (Masicampo & Lalande, 2012) and is consistent with some studies reporting non-significant effects in key tests (e.g., Celma-Miralles et al., 2016; Cirelli et al., 2016; Flaten et al., 2022; Laffere et al., 2021; Nguyen et al., 2023; Stupacher et al., 2017; Tichko et al., 2022; Zhao & Kuhl, 2020). Many other studies report key effects with p-values between *p* = .05 and *p* = .01 (e.g., (Cameron et al., 2019; Edalati et al., 2023; Gibbings et al., 2023; Lenc et al., 2018; Nave et al., 2022; Nozaradan et al., 2012; Tal et al., 2017; Vanden Bosch der Nederlanden et al., 2020) — sometimes with tests uncorrected for multiple comparisons — another sign of effects that might not replicate (Open Science Collaboration, 2015). However, several studies have been published with findings that are significant at more stringent p-values (e.g., Harding et al., 2019; Lenc et al., 2023; Mathias et al., 2020). Given the mixed evidence from various types of studies, the generally small sample sizes used, and the results of the current replication project, we argue that findings in this literature are not robust enough to support a direct relation between beat-related SSEPs and beat-related human behavior and/or perception, other than the fact that stimulus-driven activity can be recorded reliably at various frequencies that are relevant to musical rhythm and beat perception and that the stimulus-driven frequency encoding may be predicted of task-related performance. More work is necessary to fully understand the relation between frequency-tagged cortical responses and related human behaviors.

In addition to the need for conducting novel and replication studies with much larger sample sizes, one may also want to consider additional data analysis techniques that may complement or even outperform frequency-tagging in the efforts to quantify the relationship between neural activity and human beat perception. Such methods could include but are not limited to: 1) investigation of other frequency bands in the neural signal, such as via spectral analyses to estimate beat-related β-band and γ-band modulation (Fujioka et al., 2009; Snyder & Large, 2005); 2) other methods estimating synchronization of the neural signal with the stimulus envelope, such as temporal response functions (TRFs) (David et al., 2007), Phase-Locking Value (PLV) (Tal et al., 2017; Tichko et al., 2022), and canonical correlation analyses (de Cheveigné et al., 2018); 3) investigation of other event-related potentials (which may contribute to the presence of steady state responses), such as contingent negative variation (CNV) (Nobre & van Ede, 2018); 4) multivariate pattern analyses such as multiscale entropy (MSE, a measure of signal irregularity) (Bouwer et al., 2023). In addition, Bayesian statistics would be useful for potentially providing direct evidence to the contrary, (i.e., the absence of a relationship between beat-related SSEPs and beat perception and behavior (Schmalz et al., 2023), should indeed no direct relationship exist.

## Supplementary Material

1

## Figures and Tables

**Table 1.** Descriptive results and general information for each of the 14 participating labs. For each participating lab, means and standard deviations (in parentheses) are provided for the amplitude of brain activity at the frequencies of interest, as well as performance on the behavioral measures.

						Control Task					Binary Imagery						Ternary Imagery					
Lab Number	Replicating Lab	Country	Test Language	Total Tested	Total Included	0.8 Hz	1.2 Hz	1.6 Hz	2.4 Hz	Task Perf.	0.8 Hz	1.2 Hz	1.6 Hz	2.4 Hz	Task Perf.	Conf. Rating	0.8 Hz	1.2 Hz	1.6 Hz	2.4 Hz	Task Perf.	Conf. Rating
*0*	Nozaradan et al. (2011) *	-	-	*8*	*8*	*.02* *	*.004* *	*.02* *	*.23* *	-	−.002*	*.12* *	−.003*	*.2* *	-	-	*.18* *	*.004* *	*.08* *	.22*	-	-
1	braindynlab	Israel	Hebrew	17	11	−0.025 (0.034)	−0.01 (0.029)	0.001 (0.021)	0.068 (0.044)	0.977 (0.054)	−0.019 (0.032)	0.025 (0.041)	−0.003 (0.013)	0.074 (0.07)	0.702 (0.175)	4.391 (1.103)	0.002 (0.031)	0.006 (0.017)	0.01 (0.016)	0.071 (0.036)	0.526 (0.118)	4.482 (0.966)
2	capslab	USA	English	14	12	−0.004 (0.05)	0.005 (0.031)	−0.017 (0.024)	0.066 (0.033)	1.00 (0.00)	0.049 (0.078)	0.03 (0.05)	−0.015 (0.025)	0.079 (0.046)	0.658 (0.219)	5.219 (1.065)	0.016 (0.041)	0.016 (0.086)	−0.0004 (0.033)	0.067 (0.053)	0.625 (0.205)	5.106 (0.966)
3	cogsci-vassar	USA	English	15	11	−0.095 (0.322)	−0.002 (0.04)	0.052 (0.171)	0.062 (0.037)	0.97 (0.056)	−0.034 (0.093)	0.036 (0.064)	−0.009 (0.051)	0.059 (0.056)	0.716 (0.238)	5.564 (1.507)	−0.005 (0.054)	0.008 (0.03)	−0.0005 (0.024)	0.071 (0.04)	0.703 (0.242)	5.482 (1.373)
4	dvplab	United Kingdom	English	17	16	0.018 (0.024)	−0.002 (0.023)	−0.0001 (0.024)	0.035 (0.034)	0.995 (0.021)	0.014 (0.027)	0.049 (0.066)	−0.019 (0.022)	0.091 (0.08)	0.762 (0.171)	5.25 (0.903)	0.059 (0.069)	0.009 (0.033)	0.023 (0.068)	0.089 (0.115)	0.713 (0.236)	5.063 (0.701)
5	grahnlab	Canada	English	11	8	−0.001 (0.037)	−0.007 (0.015)	−0.008 (0.014)	0.059 (0.025)	0.99 (0.029)	−0.008 (0.019)	0.031 (0.029)	−0.004 (0.025)	0.09 (0.038)	0.626 (0.149)	5.112 (0.476)	0.018 (0.022)	−0.012 (0.015)	0.004 (0.019)	0.079 (0.042)	0.679 (0.186)	4.838 (0.605)
6	huettig	Netherlands	English	14	10	0.007 (0.036)	−0.012 (0.018)	−0.004 (0.013)	0.067 (0.044)	1.00 (0.00)	−0.007 (0.019)	0.018 (0.02)	0.003 (0.027)	0.076 (0.06)	0.56 (0.201)	4.90 (0.97)	0.012 (0.028)	0.002 (0.033)	0.009 (0.015)	0.065 (0.036)	0.63 (0.206)	4.91 (0.923)
7	labnpf	Portugal	Portuguese	17	9	0.002 (0.033)	0.021 (0.035)	−0.008 (0.015)	0.034 (0.037)	0.963 (0.111)	−0.022 (0.041)	0.009 (0.041)	0.007 (0.025)	0.055 (0.054)	0.544 (0.224)	4.244 (1.646)	0.039 (0.041)	−0.004 (0.025)	0.011 (0.024)	0.075 (0.056)	0.578 (0.233)	4.233 (1.71)
8	mib-au	Denmark	English	16	8	−0.017 (0.031)	−0.009 (0.026)	−0.004 (0.019)	0.041 (0.036)	1.00 (0.00)	−0.023 (0.032)	−0.007 (0.027)	0.001 (0.026)	0.047 (0.045)	0.55 (0.177)	4.471 (0.923)	0.024 (0.028)	0.005 (0.028)	0.001 (0.017)	0.046 (0.036)	0.618 (0.224)	4.487 (0.964)
9	mindlab	USA	English	16	13	0.045 (0.187)	−0.012 (0.035)	−0.006 (0.032)	0.047 (0.073)	0.936 (0.141)	−0.02 (0.094)	0.053 (0.079)	−0.01 (0.065)	0.098 (0.075)	0.738 (0.18)	5.577 (0.65)	0.069 (0.075)	0.015 (0.059)	0.017 (0.06)	0.089 (0.087)	0.792 (0.233)	5.708 (0.554)
10	bdl-uakl	New Zealand	English	16	11	−0.004 (0.061)	0.001 (0.041)	0.006 (0.02)	0.055 (0.038)	0.992 (0.025)	−0.025 (0.042)	0.09 (0.171)	0.005 (0.021)	0.107 (0.06)	0.705 (0.205)	4.964 (1.338)	0.055 (0.081)	−0.03 (0.044)	0.026 (0.071)	0.115 (0.065)	0.614 (0.247)	4.691 (1.5)
11	smartlab	Canada	English	11	10	−0.028 (0.054)	−0.039 (0.046)	−0.035 (0.081)	0.067 (0.078)	0.992 (0.026)	0.013 (0.086)	0.054 (0.047)	−0.017 (0.018)	0.118 (0.077)	0.55 (0.276)	5.16 (1.289)	0.071 (0.116)	−0.003 (0.03)	0.03 (0.092)	0.102 (0.038)	0.68 (0.22)	5.17 (1.223)
12	unlv-acnl	USA	English	23	19	−0.0004 (0.055)	−0.003 (0.033)	−0.014 (0.04)	0.085 (0.043)	0.969 (0.041)	0.008 (0.062)	0.011 (0.029)	0.006 (0.033)	0.078 (0.041)	0.632 (0.216)	5.177 (0.939)	0.011 (0.061)	0.01 (0.026)	0.005 (0.021)	0.066 (0.038)	0.581 (0.217)	5.066 (1.005)
13	keitellab	United Kingdom	English	17	14	−0.042 (0.029)	−0.009 (0.044)	−0.008 (0.021)	0.043 (0.045)	0.97 (0.062)	−0.021 (0.059)	0.043 (0.056)	−0.011 (0.026)	0.093 (0.078)	0.814 (0.146)	5.114 (0.956)	−0.007 (0.073)	−0.014 (0.019)	0.026 (0.041)	0.071 (0.069)	0.714 (0.207)	5.121 (0.937)
14^+^	vumc-mtsu	USA	English	8	4	−0.07 (0.085)	0.003 (0.024)	−0.009 (0.014)	0.042 (0.024)	1.00 (0.00)	−0.001 (0.032)	0.045 (0.036)	−0.004 (0.021)	0.044 (0.034)	0.675 (0.359)	6.075 (0.574)	0.08 (0.079)	−0.009 (0.051)	0.019 (0.054)	0.051 (0.028)	0.8 (0.082)	5.425 (0.806)

* Note: values reported for Nozaradan et al. (2011) are medians, as reported in the original paper.

+ Lab 14 excluded due to n<8 included participants.

**Table 2.** Results of Repeated Measures ANOVA for the original study and participating labs. For the Repeated Measures ANOVA applied to each lab, the main effect of Condition is reported. For the Repeated Measures ANOVA involving all 13 labs (last row, *N* = 152), the main effect of Condition and the interaction Lab x Condition are reported. Lab 0 = Nozaradan et al. (2011).

Lab Number	One-Way Repeated Measures ANOVAs by Lab							
	0.8 Hz (Ternary Frequency)		1.2 Hz (Binary Frequency)		1.6 Hz (1st Ternary Harmonic)		2.4 Hz (Stimulus Frequency)	
	*F*	*ηp* ^*2*^	*F*	*ηp* ^*2*^	*F*	*ηp* ^*2*^	*F*	*ηp* ^*2*^
0	*F*(1.8,12.4) = 12.5 ***p* = .001**	.64	*F*(1.2,13.4) = 11.5 ***p* = .008**	.62	*F*(1.2,7.9) = 22.1 ***p* = .001**	.76	*F*(1.6,11.5) = .7 ***p* = .494**	.09
1	*F*(2,20)=1.804 *p*=.19	.153	*F*(1.211,12.113)=5.342 ***p*=.034**	.348	*F*(2,20)=1.993 *p*=.165	.166	*F*(1.332,13.316)=.063 *p*=.871	.006
2	*F*(2,22)=4.115 ***p*=.030**	.272	*F*(2,22)=.433 *p*=.654	.038	*F*(2,22)=1.536 *p*=.237	.123	*F*(2,22)=.751 *p*=.484	.064
3	*F*(1.065,1.646)=.919 *p*=.366	.084	*F*(2,20)=1.581 *p*=.230	.136	*F*(1.128,11.278)=1.053 *p*=.337	.095	*F*(2,20)=.685 *p*=.515	.064
4	*F*(1.426,21.391)=5.278 ***p*=.022**	.26	*F*(1.343,2.144)=4.784 *p*=.016	.242	*F*(1.351,2.260)=3.890 *p*=.052	.206	*F*(1.182,17.726)=4.171 *p*=.051	.218
5	*F*(2,14)=2.408 *p*=.126	.256	*F*(2,14)=9.153 ***p*=.003**	.567	*F*(2,14)=.708 *p*=.509	.092	*F*(2,14)=2.659 *p*=.105	.275
6	*F*(2,18)=2.011 *p*=.163	.183	*F*(2,18)=3.295 *p*=.06	.268	*F*(2,18)=.839 *p*=.448	.085	*F*(2,18)=.677 *p*=.521	.07
7	*F*(2,16)=6.554 ***p*=.008**	.45	*F*(2,16)=.891 *p*=.429	.1	*F*(2,16)=1.662 *p*=.221	.172	*F*(2,16)=3.419 ***p*=.058**	.299
8	*F*(2,14)=6.425 *p*=.01	.479	*F*(2,14)=.506 *p*=.614	.067	*F*(1.169,8.182)=.273 *p*=.651	.037	*F*(2,14)=.147 *p*=.864	.021
9	*F*(1.247,14.962)=1.375 *p*=.269	.103	*F*(2,24)=3.340 ***p*=.053**	.218	*F*(2,24)=1.483 *p*=.247	.11	*F*(2,24)=3.079 ***p*=.094**	.204
10	*F*(2,20)=6.008 ***p*=.009**	.375	*F*(1.094,1.936)=4.496 *p*=.055	.31	*F*(1.245,12.447)=.805 *p*=.413	.074	*F*(2,20)=8.531 ***p*=.002**	.46
11	*F*(2,18)=2.947 *p*=.078	.247	*F*(2,18)=1.458 ***p*=<.001**	.537	*F*(1.243,11.183)=1.984 *p*=.187	.181	*F*(2,18)=1.321 *p*=.292	.128
12	*F*(2,36)=.303 *p*=.741	.017	*F*(2,36)=1.268 *p*=.294	.066	*F*(2,36)=1.912 *p*=.162	.096	*F*(2,36)=3.092 *p*=.058	.147
13	*F*(1.285,16.708)=1.293 *p*=.284	.09	*F*(2,26)=8.050 ***p*=.002**	.382	*F*(2,26)=6.389 ***p*=.006**	.33	*F*(2,26)=3.095 *p*=.062	.192
Lab 1:13*	Condition *F*(1.705,236.930) = 11.942 ***p*=<.001**	.041	Condition *F*(1.609,223.656)=26.356 ***p*=<.001**	.105	Condition *F*(1.715,238.400)=6.288 ***p*=.004**	.027	Condition *F*(1.873,26.394)=14.896 ***p*=<.001**	.035
	Lab x Condition *F*(2.454,236.930) = 1.079 *p*=.273	.045	Lab x Condition *F*(19.308,223.656)=1.746 ***p*=.03**	.083	Lab x Condition *F*(2.581,238.400)=1.210 *p*=.244	.062	Lab x Condition *F*(22.480,26.394)=1.525 *p*=.064	.042

* This analysis was not pre-registered and was added post- data collection.

+ Lab 14 excluded due to n<8 included participants.

**Table 3.** Results of post-hoc t-tests for the original study and participating labs. Final row indicates results when collapsing across all 13 participating labs (*N* = 152). Lab 0 = Nozaradan et al. (2011).

Lab Number	Post Hoc t-tests											
	0.8 Hz (Ternary Frequency)			1.2 Hz (Binary Frequency)			1.6 Hz (1st Ternary Harmonic)			2.4 Hz (Stimulus Frequency)		
	*C vs B*	*C vs T*	*B vs T*	*C vs B*	*C vs T*	*B vs T*	*C vs B*	*C vs T*	*B vs T*	*C vs B*	*C vs T*	*B vs T*
0	-	*t*(7)=4.1 ***p*=.005**	*t*(7)=4.0 ***p*=.005**	*t*(7)=3.4 ***p*=.012**	-	*t*(7)=3.6 ***p*=.009**	-	*t*(7)=6.1 ***p*<.001**	*t*(7)=1.8 ***p*<.001**	-	-	-
1	*t*(10)=−.416 *p*=1.00	*t*(10)=−1.813 *p*=.255	*t*(10)=−1.397 *p*=.533	*t*(10)=−3.26 ***p*=.012**	*t*(10)=−1.429 *p*=.506	*t*(10)=1.832 *p*=.246	*t*(10)=.349 *p*=1.00	*t*(10)=−1.527 *p*=.427	*t*(10)=−1.877 *p*=.226	*t*(10)=−.354 *p*=1.00	*t*(10)=−.183 *p*=1.00	*t*(10)=.171 *p*=1.00
2	*t*(11)=−2.839 ***p*=.029**	*t*(11)=−1.064 *p*=.896	*t*(11)=1.775 *p*=.269	*t*(11)=−.93 *p*=1.00	*t*(11)=−.433 *p*=1.00	*t*(11)=.496 *p*=1.00	*t*(11)=−.201 *p*=1.00	*t*(11)=−1.608 *p*=.366	*t*(11)=−1.408 *p*=.52	*t*(11)=−1.085 *p*=.869	*t*(11)=−.049 *p*=1.00	*t*(11)=1.036 *p*=.934
3	*t*(10)=−.904 *p*=1.00	*t*(10)=−1.327 *p*=.599	*t*(10)=−.423 *p*=1.00	*t*(10)=−1.711 *p*=.308	*t*(10)=−4.35 *p*=1.00	*t*(10)=1.276 *p*=.65	*t*(10)=1.338 *p*=.588	*t*(10)=1.156 *p*=.784	*t*(10)=−.182 *p*=1.00	*t*(10)=.213 *p*=1.00	*t*(10)=−.819 *p*=1.00	*t*(10)=−1.104 *p*=.849
4	*t*(15)=.258 *p*=1.00	*t*(15)=−2.676 ***p*=.036**	*t*(15)=−2.934 ***p*=.019**	*t*(15)=−2.927 ***p*=.019**	*t*(15)=−.598 *p*=1.00	*t*(15)=2.329 *p*=.08	*t*(15)=1.22 *p*=.696	*t*(15)=−1.562 *p*=.386	*t*(15)=−2.782 ***p*=.028**	*t*(15)=−2.528 *p*=.051	*t*(15)=−2.473 *p*=.058	*t*(15)=.055 *p*=1.00
5	*t*(7)=.598 *p*=1.00	*t*(7)=−1.53 *p*=.445	*t*(7)=−2.128 *p*=.155	*t*(7)=−3.443 ***p*=.012**	*t*(7)=.477 *p*=1.00	*t*(7)=3.921 ***p*=.005**	*t*(7)=−.405 *p*=1.00	*t*(7)=−1.172 *p*=.783	*t*(7)=−.766 *p*=1.00	*t*(7)=−2.28 *p*=.116	*t*(7)=−1.441 *p*=.515	*t*(7)=.839 *p*=1.00
6	*t*(9)=1.461 *p*=.484	*t*(9)=−.46 *p*=1.00	*t*(9)=−1.92 *p*=.212	*t*(9)=−2.566 *p*=.058	*t*(9)=−1.221 *p*=.714	*t*(9)=1.346 *p*=.585	*t*(9)=−.685 *p*=1.00	*t*(9)=−1.295 *p*=.635	*t*(9)=−.637 *p*=1.00	*t*(9)=−.889 *p*=1.00	*t*(9)=.206 *p*=1.00	*t*(9)=1.095 *p*=.864
7	*t*(8)=1.429 *p*=.517	*t*(8)=−2.167 *p*=.137	*t*(8)=−3.595 ***p*=.007**	*t*(8)=.658 *p*=1.00	*t*(8)=1.335 *p*=.601	*t*(8)=.678 *p*=1.00	*t*(8)=−1.41 *p*=.533	*t*(8)=−1.706 *p*=.322	*t*(8)=−.296 *p*=1.00	*t*(8)=−1.388 *p*=.553	*t*(8)=−2.613 *p*=.056	*t*(8)=−1.225 *p*=.715
8	*t*(7)=.404 *p*=1.00	*t*(7)=−2.883 ***p*=.036**	*t*(7)=−3.287 *p*=.016	*t*(7)=−.183 *p*=1.00	*t*(7)=−.948 *p*=1.00	*t*(7)=−.765 *p*=1.00	*t*(7)=−.613 *p*=1.00	*t*(7)=−.663 *p*=1.00	*t*(7)=−.049 *p*=1.00	*t*(7)=−.529 *p*=1.00	*t*(7)=−.367 *p*=1.00	*t*(7)=.162 *p*=1.00
9	*t*(12)=1.171 *p*=.76	*t*(12)=−.432 *p*=1.00	*t*(12)=−1.602 *p*=.367	*t*(12)=−2.573 ***p*=.050**	*t*(12)=−1.07 *p*=.886	*t*(12)=1.503 *p*=.438	*t*(12)=.274 *p*=1.00	*t*(12)=−1.336 *p*=.583	*t*(12)=−1.61 *p*=.362	*t*(12)=−2.33 *p*=.086	*t*(12)=−1.905 *p*=.207	*t*(12)=.425 *p*=1.00
10	*t*(10)=.874 *p*=1.00	*t*(10)=−2.468 *p*=.068	*t*(10)=−3.342 ***p*=.01**	*t*(10)=−2.156 *p*=.13	*t*(10)=.727 *p*=1.00	*t*(10)=2.883 ***p*=.028**	*t*(10)=.042 *p*=1.00	*t*(10)=−1.077 *p*=.833	*t*(10)=−1.119 *p*=.829	*t*(10)=−3.28 ***p*=.011**	*t*(10)=−3.815 ***p*=.003**	*t*(10)=−.535 *p*=1.00
11	*t*(9)=−1.001 *p*=.991	*t*(9)=−2.416 *p*=.08	*t*(9)=−1.415 *p*=.522	*t*(9)=−4.535 ***p*<.001**	*t*(9)=−1.759 *p*=.287	*t*(9)=2.777 ***p*=.037**	*t*(9)=−.533 *p*=1.00	*t*(9)=−1.929 *p*=.209	*t*(9)=−1.396 *p*=.539	*t*(9)=−1.587 *p*=.389	*t*(9)=−1.096 *p*=.863	*t*(9)=.491 *p*=1.00
12	*t*(18)=−.554 *p*=1.00	*t*(18)=−.75 *p*=1.00	*t*(18)=−.196 *p*=1.00	*t*(18)=−1.403 *p*=.507	*t*(18)=−1.354 *p*=.552	*t*(18)=.049 *p*=1.00	*t*(18)=−1.753 *p*=.264	*t*(18)=−1.627 *p*=.337	*t*(18)=. 126 *p*=1.00	*t*(18)=.85 *p*=1.00	*t*(18)=2.449 *p*=.058	*t*(18)=1.599 *p*=.356
13	*t*(13)=−.971 *p*=1.00	*t*(13)=−1.595 *p*=.368	*t*(13)=−.624 *p*=1.00	*t*(13)=−3.318 ***p*=.008**	*t*(13)=.295 *p*=1.00	*t*(13)=3.613 ***p*=.004**	*t*(13)=.251 *p*=1.00	*t*(13)=−2.963 ***p*=.019**	*t*(13)=−3.213 ***p*=.01**	*t*(13)=−2.483 *p*=.059	*t*(13)=−1.375 *p*=.542	*t*(13)=1.108 *p*=.834
Lab 1:13*	*t*(151)=−.936 *p*=1.00	*t*(151)=−5.221 ***p*=<.001**	*t*(151)=−4.285 ***p*=<.001**	*t*(151)=−6.668 ***p*=<.001**	*t*(151)=−.876 *p*=1.00	*t*(151)=5.792 ***p*=<.001**	*t*(151)=. 185 *p*=1.00	*t*(151)=−3.035 ***p*=.008**	*t*(151)=−3.22 ***p*=.004**	*t*(151)=−4.717 ***p*=<.001**	*t*(151)=−3.965 ***p*=<.001**	*t*(151)=.752 *p*=1.00

* This analysis was not pre-registered and was added post- data collection.

**Table 4.** Results of Pearson’s correlations conducted across the entire dataset and by individual lab among the following variables: amplitude (EEG) in microvolts at the binary beat frequency (1.2 Hz) during binary imagery [Binary Beat], amplitude (EEG) in microvolts at the ternary beat frequency (0.8 Hz) during ternary imagery [Ternary Beat], music training in years [Music], dance training in years [Dance], average of imagery ratings [Imagery Rating], average of accuracy on probe task [Probe Accuracy].

		Correlations						
Lab Number	Variables	*Stimulus: 2.4Hz (uV)*	*Binary: 1.2Hz (uV)*	*Ternary: 0.8Hz (uV)*	*Music (Years)*	*Dance (Years)*	*Imagery Rating*	*Probe Accuracy*
**Lab 1:13**	*Stimulus: 2.4Hz (uV)*	—						
	*Binary: 1.2Hz (uV)*	.399***	—					
	*Ternary: 0.8Hz (uV)*	.290***	.241**	—				
	*Music (Years)*	−.065	−.07	−.208*	—			
	*Dance (Years)*	.071	.123	−.063	.141	—		
	*Imagery Rating*	.166*	.080	.111	.018	.130	—	
	*Probe Accuracy*	.093	−.010	.122	.141	.059	.363***	—
**Lab 1**	*Stimulus: 2.4Hz (uV)*	—						
	*Binary: 1.2Hz (uV)*	.550	—					
	*Ternary: 0.8Hz (uV)*	.386	.125	—				
	*Music (Years)*	−.222	−.343	.104	—			
	*Dance (Years)*	.406	.383	−.125	−.011	—		
	*Imagery Rating*	.363	.165	.162	−.264	.197	—	
	*Probe Accuracy*	−.313	.262	−.449	.292	.071	−.022	—
**Lab 2**	*Stimulus: 2.4Hz (uV)*	—						
	*Binary: 1.2Hz (uV)*	−.119	—					
	*Ternary: 0.8Hz (uV)*	−.553	.273	—				
	*Music (Years)*	.446	−.411	−.094	—			
	*Dance (Years)*	−.28	−.029	−.022	.034	—		
	*Imagery Rating*	.002	−356	−.167	.220	.819**	—	
	*Probe Accuracy*	−.235	.175	.262	−.095	.548	.532	—
**Lab 3**	*Stimulus: 2.4Hz (uV)*	—						
	*Binary: 1.2Hz (uV)*	.562	—					
	*Ternary: 0.8Hz (uV)*	.667*	.423	_—_				
	*Music (Years)*	−.108	.298	−.173	—			
	*Dance (Years)*	.185	−.194	−.13	−.086	—		
	*Imagery Rating*	.575	.093	.517	−.485	.144	—	
	*Probe Accuracy*	.465	.111	.465	−.245	−.110	.771**	—
**Lab 4**	*Stimulus: 2.4Hz (uV)*	—						
	*Binary: 1.2Hz (uV)*	.733**	—					
	*Ternary: 0.8Hz (uV)*	.26	.367	—				
	*Music (Years)*	−.261	−.263	−.299	—			
	*Dance (Years)*	.351	.568*	.104	.115	—		
	*Imagery Rating*	.210	.191	−.146	−.034	−.134	—	
	*Probe Accuracy*	−.14	−.044	.389	.029	.061	.302	—
**Lab 5**	*Stimulus: 2.4Hz (uV)*	—						
	*Binary: 1.2Hz (uV)*	−.161	—					
	*Ternary: 0.8Hz (uV)*	.069	.763*	—				
	*Music (Years)*	−.133	−.713*	−.547	—			
	*Dance (Years)*	.019	−.576	−.147	.182	—		
	*Imagery Rating*	.326	−.508	−.052	−.059	.751*	—	
	*Probe Accuracy*	.560	.169	.492	−.123	−.201	.102	—
**Lab 6**	*Stimulus: 2.4Hz (uV)*	—						
	*Binary: 1.2Hz (uV)*	−.660*	—					
	*Ternary: 0.8Hz (uV)*	.314	.087	—				
	*Music (Years)*	.752*	−.708*	.0002	—			
	*Dance (Years)*	.235	−.169	.338	.13	—		
	*Imagery Rating*	−.336	.088	.135	−.584	.058	—	
	*Probe Accuracy*	.125	−.118	.413	−.056	−.492	.426	—
**Lab 7**	*Stimulus: 2.4Hz (uV)*	—						
	*Binary: 1.2Hz (uV)*	−.238	—					
	*Ternary: 0.8Hz (uV)*	−.003	.029	—				
	*Music (Years)*	.002	−.500	−.329	—			
	*Dance (Years)*	−.395	−.103	−.087	.521	—		
	*Imagery Rating*	−.230	.061	.271	.492	.370	—	
	*Probe Accuracy*	.210	−.403	.197	.778*	.383	.583	—
**Lab 8**	*Stimulus: 2.4Hz (uV)*	—						
	*Binary: 1.2Hz (uV)*	−.138	—					
	*Ternary: 0.8Hz (uV)*	−.552	.115	—				
	*Music (Years)*	.258	−.420	−.399	—			
	*Dance (Years)*	−.399	.554	.339	−.537	—		
	*Imagery Rating*	.628	−.169	.079	.269	−.490	—	
	*Probe Accuracy*	−.298	−.222	.418	.219	−.396	.027	—
**Lab 9**	*Stimulus: 2.4Hz (uV)*	—						
	*Binary: 1.2Hz (uV)*	−.043	—					
	*Ternary: 0.8Hz (uV)*	.219	.691**	—				
	*Music (Years)*	−.582*	−.286	−.216	—			
	*Dance (Years)*	−.190	.523	.072	−.297	—		
	*Imagery Rating*	.306	.157	.353	−.314	−.039	—	
	*Probe Accuracy*	.564*	−.371	.170	−.020	−.258	.240	—
**Lab 10**	*Stimulus: 2.4Hz (uV)*	—						
	*Binary: 1.2Hz (uV)*	.751**	—					
	*Ternary: 0.8Hz (uV)*	.632*	.13	—				
	*Music (Years)*	−.083	.336	−.442	—			
	*Dance (Years)*	−.301	−.184	−.069	−.137	—		
	*Imagery Rating*	.181	.193	.328	.244	.277	—	
	*Probe Accuracy*	−.101	−.151	.221	−.040	.532	.315	—
**Lab 11**	*Stimulus: 2.4Hz (uV)*	—						
	*Binary: 1.2Hz (uV)*	.409	—					
	*Ternary: 0.8Hz (uV)*	.334	−.021	—				
	*Music (Years)*	−.24	−.075	−.381	—			
	*Dance (Years)*	.100	−.082	−.312	.272	—		
	*Imagery Rating*	−.589	−.486	−.573	−.05	−.273	—	
	*Probe Accuracy*	−.164	−.404	−.195	.044	−.006	.572	—
**Lab 12**	*Stimulus: 2.4Hz (uV)*	—						
	*Binary: 1.2Hz (uV)*	−.017	—					
	*Ternary: 0.8Hz (uV)*	.023	−.298	—				
	*Music (Years)*	−.124	−.081	−.155	—			
	*Dance (Years)*	—	—	—	—	—		
	*Imagery Rating*	.180	−.135	.290	.175	—	—	
	*Probe Accuracy*	.452	−.101	−115	.418	—	−.247	—
**Lab 13**	*Stimulus: 2.4Hz (uV)*	**—**						
	*Binary: 1.2Hz (uV)*	.496	—					
	*Ternary: 0.8Hz (uV)*	.393	.102	—				
	*Music (Years)*	−.122	−.005	−.119	—			
	*Dance (Years)*	.031	.191	−.358	.505	—		
	*Imagery Rating*	.213	−.232	.445	.121	.161	—	
	*Probe Accuracy*	.143	.283	.151	.152	.417	.066	—

+ Note that correlations conducted with imagery ratings and probe accuracy were conducted within each respective task, such that binary beat was correlated with probe accuracy and imagery success ratings from the binary task only (and vice versa for ternary beat). In correlations with music, dance, or with each other, the grand average of imagery ratings and probe accuracy were used.

## Footnotes

1 In the original study (Nozaradan et al., 2011), beat perception is defined as “the ability to perceive periodicities from sounds that are not necessarily periodic in reality,” similar to our definition here. However, the original study also referred to 2.4 Hz as the ‘beat frequency,’ even though 2.4 Hz is in fact the stimulus periodicity, and thus a response at that frequency reflects the physical stimulus and does not necessarily entail any perceptual inference. By contrast, the imagery task requires listeners to infer a single beat level that occurs every two (or three) stimulus events. We argue that meter perception would entail inferring multiple hierarchical beat levels simultaneously (for example the ternary or binary beat pattern plus the downbeat of the measure). However, in this task listeners are only required to infer one beat level. For this reason, in this paper we refer to 2.4 Hz as the stimulus-related frequency and 0.8Hz/1.2Hz as the beat- or imagery- related frequencies.

2 Note the original study reported a One-Way ANOVA as their primary analysis, followed up by post-hoc testing. While this RR reports these results from each individual lab in a table, the meta-analyses were conducted on raw mean differences of the amplitude of electric activity at the imagery-related frequencies among the three conditions.

3 Note that labs could choose to contribute a half sample of eight participants, if necessary. Some labs who originally committed to a full sample (*n* = 16) changed to a half sample (*n* = 8) after the COVID-19 Pandemic (see Appendix A for individual lab details)

4 In the case that a lab had approval from their ethics board to run emancipated minors under the age of 18, this was allowed

5 Prior to conducting the RR study, the parent lab conducted a pilot behavioral experiment to test the training instructions and listeners’ performance on the two behavioral measures. Sixteen adult listeners with normal hearing participated in a pilot study. All participants passed training and completed 10 test trials for both binary imagery and ternary imagery conditions. Participant accuracy on the probe task (M = 0.67, SD = 0.18) was significantly above chance (t(15) = 3.709, p = .002), with no effect of imagery type or probe type and no interactions (p > .05). Their imagery success ratings (M = 5.24, SD = 1.37) were significantly above the scale midpoint (t(15) = 3.608, p = .003), with no effect of imagery type or probe type and no interactions (p > .05). While probe task performance and imagery success ratings demonstrated a positive correlation, it was not statistically significant (r = 0.41, p = .119).

6 Lab 15 was excluded prior to data analysis due to errors in their trial event code timings that could not be resolved by the first author nor the lab authors, at which point they requested their data be excluded.

7 Of the 16 participants included for Lab 4, five had their EEG data collected with a sampling frequency of 500Hz. These participants were processed with the steps that follow, except note the following differences: frequency resolution = 0.06 Hz, frequency spectrum: 0 to 250 Hz, bins for subtraction ranged from ±0.18 to ±0.30 Hz. When sampling frequency (500 Hz, 1000 Hz) was entered as a between-subjects variable in Lab 4’s One-Way ANOVAs (section 5.3), it resulted in no significant interactions with or main effects of sampling frequency. Thus, data were collapsed across sampling frequency for all subsequent analyses.
