# Supplementary material for: Registered Report: Replication and Extension of Nozaradan, Peretz, Missal and Mouraux (2011)
Source: bioRxiv. 2025 Mar 17:2025.03.13.643168. Preprint. [Version 1] doi: 10.1101/2025.03.13.643168 (PMC11956986; doi:10.1101/2025.03.13.643168)
Supplement: 1 [file NIHPP2025.03.13.643168V1-supplement-1.pdf]

bioRxiv preprint doi: https://doi.org/10.1101/2025.03.13.643168; this version posted March 17, 2025. The copyright holder for this preprint (which was not certified by peer review) is the author/funder, who has granted bioRxiv a license to display the preprint in perpetuity. It is made available under aCC-BY 4.0 International license.

Supplementary Figures

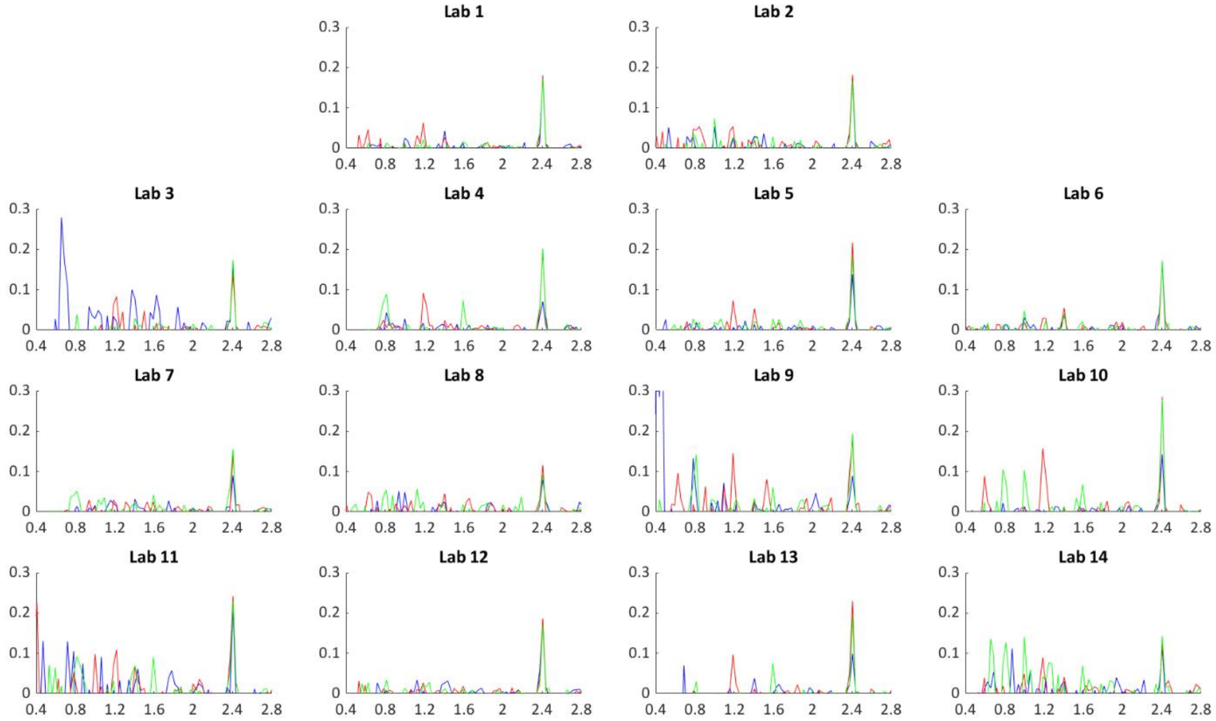

Figure S1. Averages of the stimulus- and beat- related steady-state EPs elicited by the 2.4 Hz auditory beat in the control condition (blue), the binary beat imagery condition (red), and the ternary beat imagery condition (green) for all participating labs. The frequency spectra represent the amplitude of the EEG signal (in microvolts) as a function of frequency, averaged across all scalp electrodes, after applying the noise subtraction procedure.

bioRxiv preprint doi: https://doi.org/10.1101/2025.03.13.643168; this version posted March 17, 2025. The copyright holder for this preprint (which was not certified by peer review) is the author/funder, who has granted bioRxiv a license to display the preprint in perpetuity. It is made available under aCC-BY 4.0 International license.

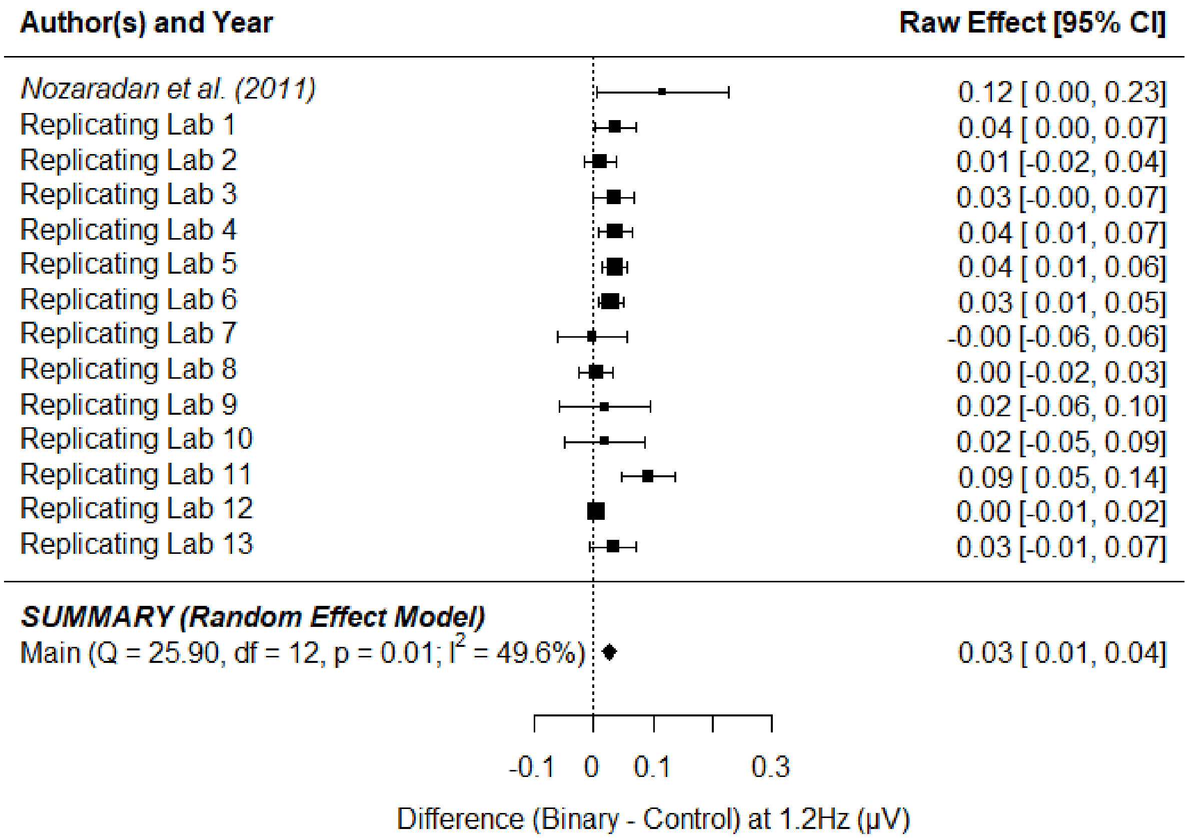

Figure S2. Point-estimate for the meta-analytic effect of condition (control task vs. binary imagery) on the binary frequency using medians. Squares indicate each lab's median difference, where the size of the square corresponds to the inverse of the standard error of the difference score, and the error bars indicate 95% confidence intervals (CI) around the median difference. Diamonds indicate the random-effects meta-analytic effect size estimate, where the width represents the 95% CI. The diamond represents the meta-analytic estimate (Note: this estimate does not include the original Nozaradan et al. (2011) result.

bioRxiv preprint doi: https://doi.org/10.1101/2025.03.13.643168; this version posted March 17, 2025. The copyright holder for this preprint (which was not certified by peer review) is the author/funder, who has granted bioRxiv a license to display the preprint in perpetuity. It is made available under aCC-BY 4.0 International license.

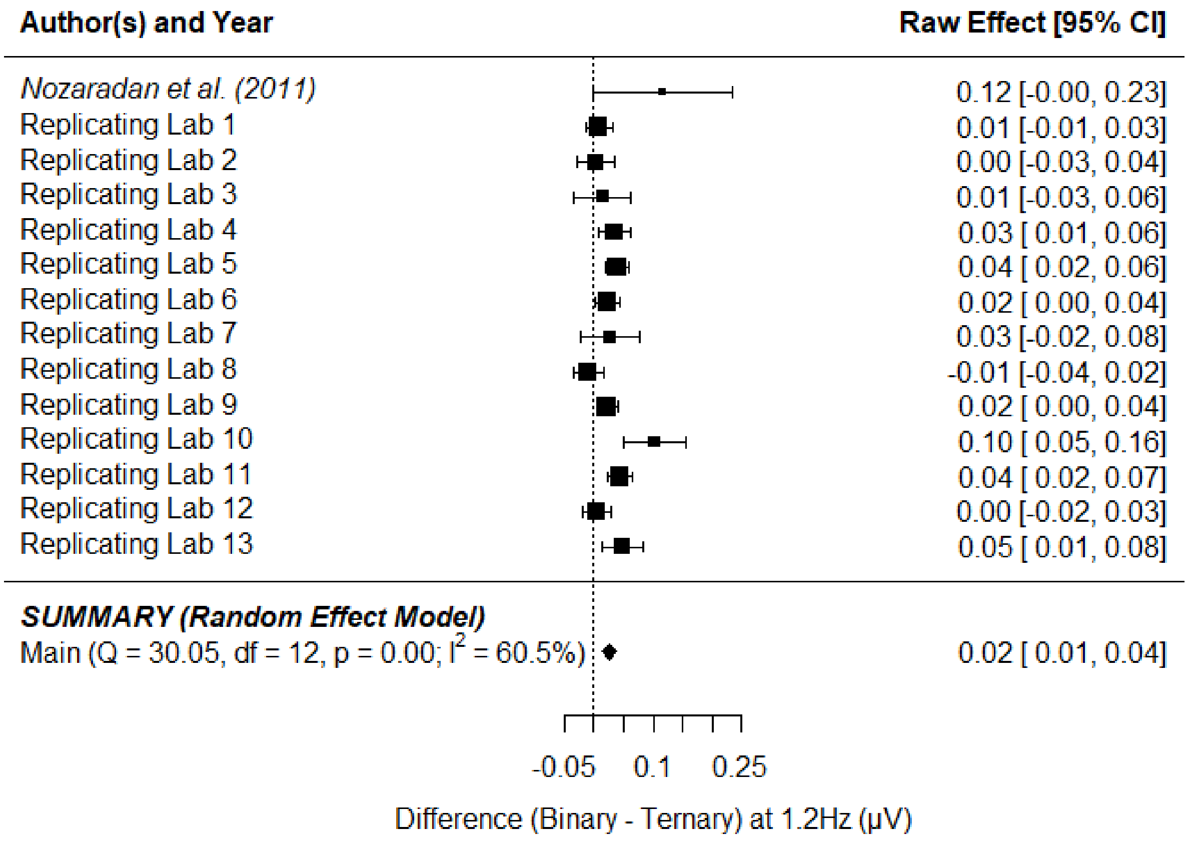

Figure S3. Point-estimate for the meta-analytic effect of condition (binary imagery vs. ternary imagery) on the binary frequency using medians. Squares indicate each lab's median difference, where the size of the square corresponds to the inverse of the standard error of the difference score, and the error bars indicate 95% confidence intervals (CI) around the median difference. Diamonds indicate the random-effects meta-analytic effect size estimate, where the width represents the 95% CI. The diamond represents the meta-analytic estimate (Note: this estimate does not include the original Nozaradan et al. (2011) result.

bioRxiv preprint doi: https://doi.org/10.1101/2025.03.13.643168; this version posted March 17, 2025. The copyright holder for this preprint (which was not certified by peer review) is the author/funder, who has granted bioRxiv a license to display the preprint in perpetuity. It is made available under aCC-BY 4.0 International license.

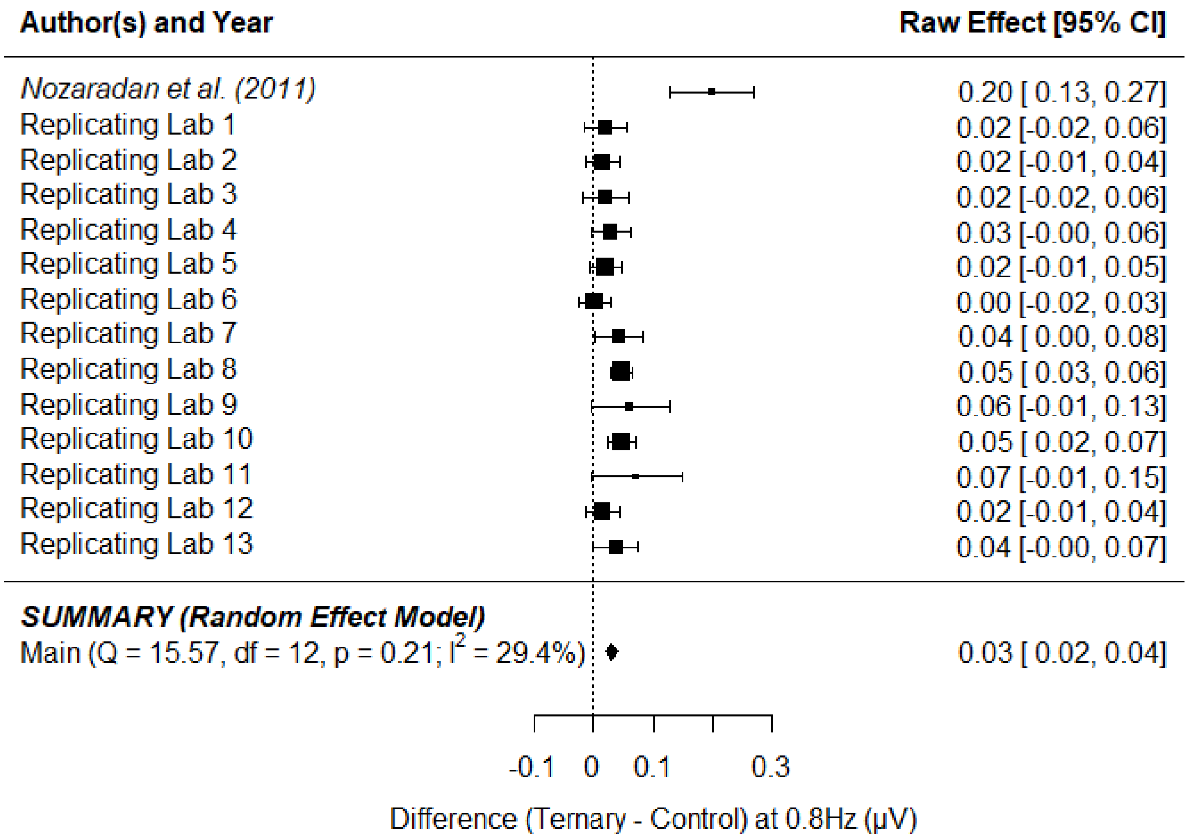

Figure S4. Point-estimate for the meta-analytic effect of condition (control task vs. ternary imagery) on the ternary frequency using medians. Squares indicate each lab's median difference, where the size of the square corresponds to the inverse of the standard error of the difference score, and the error bars indicate 95% confidence intervals (CI) around the median difference. Diamonds indicate the random-effects meta-analytic effect size estimate, where the width represents the 95% CI. The diamond represents the meta-analytic estimate (Note: this estimate does not include the original Nozaradan et al. (2011) result.

bioRxiv preprint doi: https://doi.org/10.1101/2025.03.13.643168; this version posted March 17, 2025. The copyright holder for this preprint (which was not certified by peer review) is the author/funder, who has granted bioRxiv a license to display the preprint in perpetuity. It is made available under aCC-BY 4.0 International license.

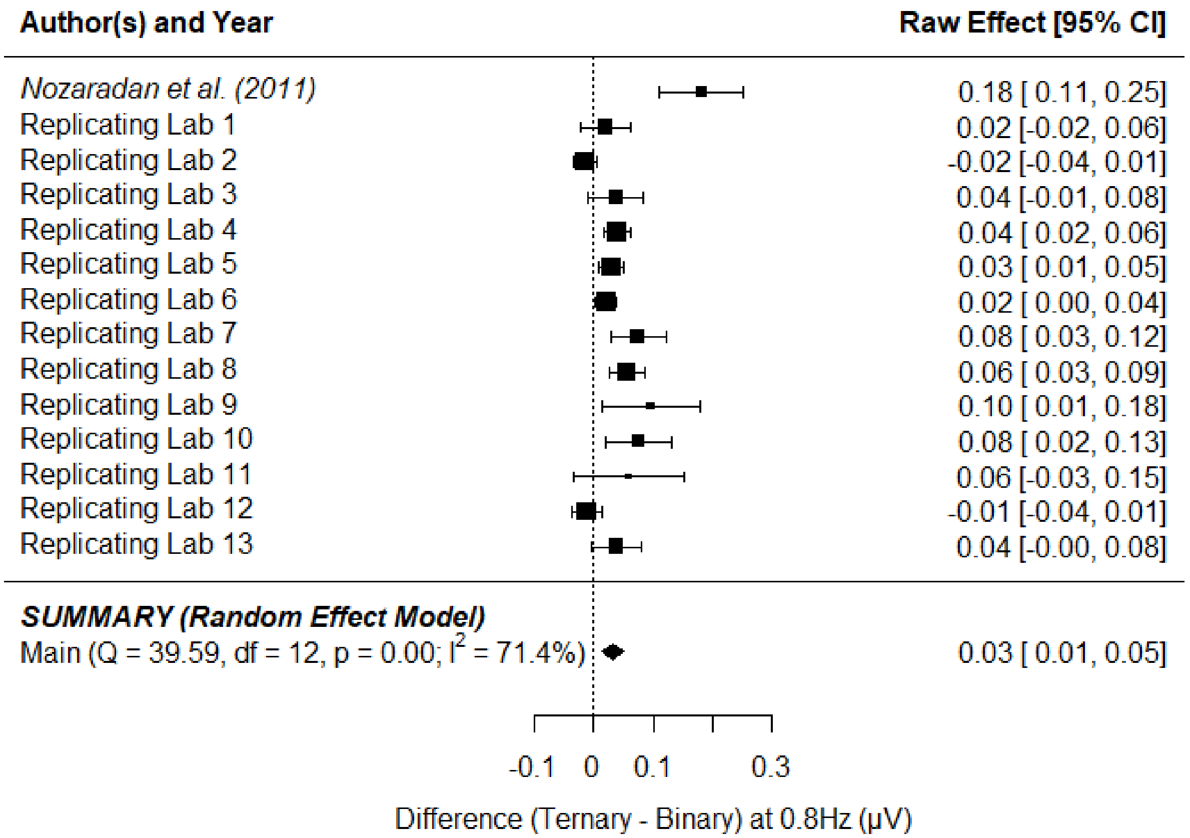

Figure S5. Point-estimate for the meta-analytic effect of condition (ternary imagery vs. binary imagery) on the ternary frequency using medians. Squares indicate each lab's median difference, where the size of the square corresponds to the inverse of the standard error of the difference score, and the error bars indicate 95% confidence intervals (CI) around the median difference. Diamonds indicate the random-effects meta-analytic effect size estimate, where the width represents the 95% CI. The diamond represents the meta-analytic estimate (Note: this estimate does not include the original Nozaradan et al. (2011) result.

Lab Number	Lab Contributors	Data Collection Location	Experiment Script	Audio Presentation	EEG System	# scalp electrodes	Additional Electrodes	EEG Sampling Rate	Total Tested	Total Included	Exclusions
1	Elana Zion Golumbic, Paz Har-Shai, Danna Pinto	Ramat Gan, Israel	Psychopy	Etymotic ER-1 ear inserts	Biosemi	64	eye/face electrodes (6); mastoids (2)	1024	17	11	6 failed training
2	Mara Breen, Ahren Fitzroy, Meg Renzelman	South Hadley, MA, USA	Presentation	JBL 104 Speakers	Biosemi	64	eye/face electrodes (6); mastoids (2)	2048 (DS to 1024)	14	12	1 failed training, 1 experiment error
3	Joshua de Leeuw, Emma Alexandrov, Dingyi Pan, Ningyao Geng, Kiara Holm	Poughkeepsie, NY, USA	Presentation	Headphones (Edifier H840)	EGI	128	None	1000	15	11	2 failed training, 2 excessive EEG artifacts
4	Christian Keitel, Lucy Wight	Stirling, UK	Psychtoolbox	Headphones (Sennheiser HD 25-1 II)	ANTNeuro Eego Sports	30	mastoid electrodes (2)	500/1000	17	16	1 rhythmic movement observed
5	Jessica Grahn, Chu Yi Yu, Karli Nave, Thibault Chabin	London, ON, Canada	Presentation	Headphones (Sennheiser HD 25-1 II)	Biosemi	64	eye/face electrodes (6); mastoids (2)	1024	11	8	3 failed training
6	Falk Huettig, Markus Ostarek, Eva Poort, Yiguang Liu	Nijmegen, Netherlands	Presentation	Speakers (JBL Cinema 5.1)	BrainVision	28	eye/face electrodes (4)	1000	14	10	2 experimenter error, 2 failed training
7	Mariana R. Pereira, Rita Pasion, Fernando Barbosa, Fernando Ferreira-Santos, Inês Macedo	Porto, Portugal	Presentation	Headphones (Sennheiser HD 206)	Netstation	128	None	1000	17	9	4 failed training, 1 experimenter error, 3 excessive EEG artifacts
8	Cecilie Møller ,Jan Stupacher, Alexandre Celma-Miralles	Aarhus, Denmark	Presentation	Etymotic ER-2 ear inserts	Brain Products	28	2 eye/face electrodes; SDM muscle (neck), FDI muscle (hand)	1000	16	8	8 failed training
9	Parker Tichko, Psyche Loui	Boston, MA, USA	Psychopy	Headphones (Sennheiser HD 280)	BrainVision	63	None	5000 (DS to 1000)	16	13	2 failed training, 1 excessive EEG artifacts
10	David Moreau, Grace Lourie	Auckland, New Zealand	Psychtoolbox	Etymotic ER-3 ear inserts	Netstation	128	None	1000	16	11	5 failed training

11	Sean A. Gilmore, Frank A. Russo	Toronto, Canada	Presentation	3M E-A-RTONE Gold 3A ear inserts	Biosemi	128	eye/face electrodes (6); mastoids (2)	1024	11	10	1 excessive EEG artifacts
12	Karli Nave, Erin Hannon, Joel Snyder	Las Vegas, NV, USA	Presentation	Etymotic ER-3 ear inserts	Biosemi	64	eye/face electrodes (6); mastoids (2)	1024	23	19	4 failed training
13	Anne Keitel & Sarah C. Allen	Dundee, UK	Psychtoolbox	Headphones (Sennheiser HD 25)	Biosemi	32	eye/face electrodes (6); mastoids (2)	1024	17	14	3 failed training
14	Eniko Ladanyi, Srishti Nayak, Reyna Gordon, Cyrille Magne	Nashville, TN, USA	E-Prime 2.0	Headphones (Bose QuietComfort 35)	EGI	28	eye/face electrodes (4)	1000	8	4	3 failed training, 1 excessive EEG artifacts

Table S1. Individual lab details for all contributing labs.

bioRxiv preprint doi: https://doi.org/10.1101/2025.03.13.643168; this version posted March 17, 2025. The copyright holder for this preprint (which was not certified by peer review) is the author/funder, who has granted bioRxiv a license to display the preprint in perpetuity. It is made available under aCC-BY 4.0 International license.

Appendix A. Individual Lab Details

See below for each lab's list of contributing authors, funding acknowledgements, and pre-registration details[8]. Pre-registration details reflect each lab's committed pre-registration plan prior to collecting data.

Lab 1: braindynlab-biu

Contributing Authors: Paz Har-Shai Yahav[1], Danna Pinto[1], Elana M. Zion Golumbic[1]
[1] The Gonda Center for Multidisciplinary Brain Research, Bar Ilan University, Ramat Gan, Israel

Funding Acknowledgements:
Israel Science Foundation Grant (ISF #2339/20)

Pre-Registration

I. Participants:

- **Stopping Criteria:** When we reach our sample size commitment
- **Sample Size Commitment:** Full block of 16, and then will stop
- **Compensation:** Course credit (1 credit per hour)
- **Normal Hearing:** Verified via self-report

II. Experiment Set-Up:

- **Testing Language:** Hebrew* (*translated by P. Har-Shai*)
- **Experiment Room:** Sound-attenuated booth (single-walled)
- **Location of Training Experimenter During Experiment:** In the same room/booth as the participant during the entire study
- **Location of Data Experimenter During Experiment:** In a separate area/room outside of the testing booth during the entire study
- **Experiment Script to be Used:** Psychopy script (written by P. Tichko)

III. Audio Delivery During Experiment:

[8] Other lab-specific information is provided in Table S1, and thus is not listed in this appendix. This includes: lab location, experiment script version, audio presentation method, EEG system, number of scalp electrodes, external (additional) electrode positions, sampling frequency (SF), low pass (LP) filter, number participants collected, number participants included (after exclusions), and exclusion reasons.

bioRxiv preprint doi: https://doi.org/10.1101/2025.03.13.643168; this version posted March 17, 2025. The copyright holder for this preprint (which was not certified by peer review) is the author/funder, who has granted bioRxiv a license to display the preprint in perpetuity. It is made available under aCC-BY 4.0 International license.

- **Training Experimenter:** Sennheiser 280 Pro headphones (experimenter removed these during test trials)
- **Participant:** Ear inserts (Etymotic ER-1; dB levels not measured)

IV. Changes in Pre-registration Due to COVID-19 Pandemic:

- **Data Collection Completed:** February 20, 2020

The lab's pre-registration was followed in all other respects.

Lab 2: capslab-mhc

Contributing Authors: Mara Breen[1], Ahren B. Fitzroy[1], Meg Renzelman[1]
[1] Mount Holyoke College, South Hadley, MA, United States

Funding Acknowledgements: James S. McDonnell Foundation Scholar Award in Understanding Human Cognition

Pre-Registration

I. Participants:

- **Stopping Criteria:** When the official time for data collection ends (i.e., will continue testing as many participants as possible until data collection closes or the specified alternative stopping date)
- **Sample Size Commitment:** Full block of 16, but will continue collecting until the end date for data collection
- **Compensation:** Course credit (1 credit)
- **Normal Hearing:** Hearing tests conducted using an audiometer

II. Experiment Set-Up:

- **Testing Language:** English
- **Experiment Room:** Room with sound-proofing materials (e.g., sound-attenuating walls, curtains, etc.)
- **Location of Training Experimenter During Experiment:** In the same room/booth as the participant to ensure compliance
- **Location of Data Experimenter During Experiment:** In a separate control room or outside the testing booth
- **Experiment Script to be Used:** Presentation script

III. Audio Delivery During Experiment:

bioRxiv preprint doi: https://doi.org/10.1101/2025.03.13.643168; this version posted March 17, 2025. The copyright holder for this preprint (which was not certified by peer review) is the author/funder, who has granted bioRxiv a license to display the preprint in perpetuity. It is made available under aCC-BY 4.0 International license.

- **Training Experimenter:** Speakers (listened to masking music through headphones during test trials)
- **Participant:** Ear inserts (Etymotic ER-3; dB levels not measured)

IV. Changes in Pre-registration Due to COVID-19 Pandemic:

- **Data Collection Completed:** March 6, 2020
- The lab's pre-registration was followed in all other respects.

Lab 3: cogsci-vassar

Contributing Authors: Emma Alexandrov[1], Joshua R. de Leeuw[1], Ningyao Geng[1], Kiara Holm[1], Dingyi Pan[1]
[1] Department of Cognitive Science, Vassar College, Poughkeepsie, NY, United States

Funding Acknowledgements: None

Pre-Registration

I. Participants:

- **Stopping Criteria:** When the specific number of participants listed below is tested, accounting for exclusions prior to data review
- **Sample Size Commitment:** Full block of 16, and will stop after reaching 16
- **Compensation:** Not specified
- **Normal Hearing:** Verified via self-report

II. Experiment Set-Up:

- **Testing Language:** English
- **Experiment Room:** Quiet location in the building
- **Location of Training Experimenter During Experiment:** In the same room/booth as the participant to ensure compliance
- **Location of Data Experimenter During Experiment:** In a separate control room or outside the testing booth
- **Experiment Script to be Used:** Presentation script

III. Audio Delivery During Experiment:

- **Training Experimenter:** Headphones (Edifier H840; experimenter removed these during test trials)
- **Participant:** Headphones (Edifier H840; dB levels not measured)

bioRxiv preprint doi: https://doi.org/10.1101/2025.03.13.643168; this version posted March 17, 2025. The copyright holder for this preprint (which was not certified by peer review) is the author/funder, who has granted bioRxiv a license to display the preprint in perpetuity. It is made available under aCC-BY 4.0 International license.

IV. Changes in Pre-registration Due to COVID-19 Pandemic:

- **Data Collection Completed:** November 23 (year unspecified)
- **Compensation:** Participants were paid $10/hour

The lab's pre-registration was followed in all other respects.

Lab 4: dvplab-ustir

Contributing Authors: Christian Keitel[1], Lucy Wight[2]
[1] Department of Psychology, University of Dundee, Dundee, UK
[2] Department of Psychology, University of Stirling, Stirling, UK

Funding Acknowledgements:
RSE Saltire Facilitation Network Award (Reference Number 1963)

Pre-Registration

I. Participants:

- **Stopping Criteria:** When the specific number of participants listed below is tested, accounting for exclusions prior to data review
- **Sample Size Commitment:** Full block of 16, and will stop after reaching 16
- **Compensation:** Financial compensation (£7.50/hour) or course credit (2 "tokens")
- **Normal Hearing:** Verified via self-report only

II. Experiment Set-Up:

- **Testing Language:** English
- **Experiment Room:** Room with occasional mild/moderate noise from adjacent rooms
- **Location of Training Experimenter During Experiment:** In the same room/booth as the participant to ensure compliance
- **Location of Data Experimenter During Experiment:** In a separate control room or outside the testing booth
- **Experiment Script to be Used:** Psychtoolbox

III. Audio Delivery During Experiment:

- **Training Experimenter:** Headphones (Philips FX3BK/00; experimenter removed these during test trials)
- **Participant:** Headphones (Sennheiser HD 25-1 II; dB levels measured: 37 dB in silence, 40.5 dB to sound)

bioRxiv preprint doi: https://doi.org/10.1101/2025.03.13.643168; this version posted March 17, 2025. The copyright holder for this preprint (which was not certified by peer review) is the author/funder, who has granted bioRxiv a license to display the preprint in perpetuity. It is made available under aCC-BY 4.0 International license.

IV. Changes in Pre-registration Due to COVID-19 Pandemic:

- **Data Collection Completed:** December 2, 2021
- **Location of Training Experimenter During Experiment:** Due to COVID-19 guidance, the training experimenter only stayed for the practice and monitored compliance through a window.

The lab's pre-registration was followed in all other respects.

Lab 5: grahnlab

Contributing Authors: Thibault Chabin[1], Jessica A. Grahn[1], Karli M. Nave[1,2], Chu Yi Yu[1]
[1] Department of Psychology, Centre for Brain and Mind, University of Western Ontario, London, ON, Canada
[2] Department of Psychology, University of Nevada Las Vegas, Las Vegas, NV, United States

Funding Acknowledgements: Discovery Grant and Steacie Award from NSERC to JAG, NSERC-CREATE Complex Dynamics Fellowship (to KMN)

Pre-Registration

I. Participants:

- **Stopping Criteria:** When the specific number of participants listed below is tested, accounting for exclusions prior to data review
- **Sample Size Commitment:** Full block of 16, and will stop after reaching 16
- **Compensation:** $10/hour or 1 credit/hour or no financial compensation with course credit
- **Normal Hearing:** Verified via self-report only

II. Experiment Set-Up:

- **Testing Language:** English
- **Experiment Room:** Sound-"proof" booth (single-walled)
- **Location of Training Experimenter During Experiment:** In the same room/booth as the participant to ensure compliance
- **Location of Data Experimenter During Experiment:** In a separate control room or outside the testing booth
- **Experiment Script to be Used:** MATLAB

III. Audio Delivery During Experiment:

bioRxiv preprint doi: https://doi.org/10.1101/2025.03.13.643168; this version posted March 17, 2025. The copyright holder for this preprint (which was not certified by peer review) is the author/funder, who has granted bioRxiv a license to display the preprint in perpetuity. It is made available under aCC-BY 4.0 International license.

- **Training Experimenter:** Headphones (Sennheiser HD 25-1 II; experimenter removed these during test trials)
- **Participant:** Headphones (Sennheiser HD 25-1 II; dB levels measured: 30.6 dB in silence, 84.6 dB to sound using standardized testing)

IV. Changes in Pre-registration Due to COVID-19 Pandemic:

- **Data Collection Completed:** April 29, 2022
- **Compensation:** All participants were paid $10/hour
- **Experiment Script to be Used:** Presentation

The lab's pre-registration was followed in all other respects.

Lab 6: huettig-mpi

Contributing Authors: Falk Huettig[1], Yiguang Liu[2], Markus Ostarek[1], Eva D. Poort[1]
[1] Max Planck Institute for Psycholinguistics, Nijmegen, The Netherlands
[2] School of International Studies, Zhejiang University, Hangzhou, China

Funding Acknowledgements: None

Pre-Registration

I. Participants:

- **Stopping Criteria:** When the specific number of participants listed below is tested, accounting for exclusions prior to data review
- **Sample Size Commitment:** Full block of 16, and will stop after reaching 16
- **Compensation:** Not specified
- **Normal Hearing:** Verified via self-report only

II. Experiment Set-Up:

- **Testing Language:** English
- **Experiment Room:** Sound-"proof" booth (single-walled)
- **Location of Training Experimenter During Experiment:** In the same room/booth as the participant to ensure compliance
- **Location of Data Experimenter During Experiment:** In a separate control room or outside the testing booth
- **Experiment Script to be Used:** Presentation

III. Audio Delivery During Experiment:

bioRxiv preprint doi: https://doi.org/10.1101/2025.03.13.643168; this version posted March 17, 2025. The copyright holder for this preprint (which was not certified by peer review) is the author/funder, who has granted bioRxiv a license to display the preprint in perpetuity. It is made available under aCC-BY 4.0 International license.

- **Training Experimenter:** Speakers (JBL Cinema 5.1; experimenter listened to masking music through headphones during testing)
- **Participant:** Speakers (JBL Cinema 5.1; dB levels not measured)

IV. Changes in Pre-registration Due to COVID-19 Pandemic:

- **Data Collection Completed:** October 14, 2020
- **Compensation:** Participants were paid €10/hour

The lab's pre-registration was followed in all other respects.

Lab 7: labnpf-up

Contributing Authors: Fernando Barbosa[1], Fernando Ferreira-Santos[2], Rita Pasion[2], Mariana R. Pereira[1], Inês Macedo[1]
[1] Laboratory of Neuropsychophysiology, Faculty of Psychology and Education Sciences, University of Porto, Porto, Portugal
[2] HEI-Lab: Digital Human-Environment Interaction Labs, Universidade Lusófona, Porto, Portugal

Funding Acknowledgements: FUNDAÇÃO PARA A CIÊNCIA E TECNOLOGIA (FCT), UNDER HEI-LAB R&D UNIT (UIDB/05380/2020)

Pre-Registration

I. Participants:

- **Stopping Criteria:** When I have tested the specific number of participants listed below, accounting for exclusions prior to looking at the data.
- **Sample Size Commitment:** Full block of 16, and we will stop after we reach 16.
- **Compensation:** Financial compensation.
- **Normal Hearing:** Relied on self-report only.

II. Experiment Set-Up:

- **Testing Language:** Portuguese (translated by ?).
- **Experiment Room:** Room with sound-proofing materials (e.g., sound attenuating walls, curtains, etc.).
- **Location of Training Experimenter During Experiment:** In the same room/booth as the participant, and they will remain there throughout the experiment to ensure participant compliance with instructions.
- **Location of Data Experimenter During Experiment:** In a separate control room or outside the testing booth for the entire experiment.

bioRxiv preprint doi: https://doi.org/10.1101/2025.03.13.643168; this version posted March 17, 2025. The copyright holder for this preprint (which was not certified by peer review) is the author/funder, who has granted bioRxiv a license to display the preprint in perpetuity. It is made available under aCC-BY 4.0 International license.

- **Experiment Script to be Used:** Presentation.

III. Audio Delivery During Experiment:

- **Training Experimenter:** Headphones (Sennheiser HD 206; experimenter removed these during test trials).
- **Participant:** Headphones (Sennheiser HD 206; dB were measured; 35-47 dB in silence).

IV. Changes in Pre-registration Due to COVID Pandemic:

- **Data collection completed:** N/A.
- **Compensation:** Participants were compensated with a 10€ shopping voucher.
- **dB in Silence:** 32.1 dB was measured (October 8th, 2021).
- **dB to Sound:** Record of measurement missing.

The lab's pre-registration was followed in all other respects.

Lab 8: mib-au

Contributing Authors: Alexandre Celma-Miralles[1], Cecilie Møller[1], Jan Stupacher[1]
[1] Department of Clinical Medicine – Center for Music in the Brain, Aarhus University & The Royal Academy of Music, Aarhus, Denmark

Funding Acknowledgements: CM and JS were supported by Seed Funding from the Interacting Minds Centre, Aarhus University (2019-128). The Center for Music in the Brain is funded by the Danish National Research Foundation (DNRF 117).

Pre-Registration

I. Participants:

- **Stopping Criteria:** When I have tested the specific number of participants listed below, accounting for exclusions prior to looking at the data.
- **Sample Size Commitment:** Full block of 16, and we will stop after we reach 16.
- **Compensation:** Financial compensation (225 DKK).
- **Normal Hearing:** Relied on self-report only.

II. Experiment Set-Up:

- **Testing Language:** English.
- **Experiment Room:** Room with sound-proofing materials (e.g., sound attenuating walls, curtains, etc.).

bioRxiv preprint doi: https://doi.org/10.1101/2025.03.13.643168; this version posted March 17, 2025. The copyright holder for this preprint (which was not certified by peer review) is the author/funder, who has granted bioRxiv a license to display the preprint in perpetuity. It is made available under aCC-BY 4.0 International license.

- **Location of Training Experimenter During Experiment:** In the same room/booth as the participant, and they will remain there throughout the experiment to ensure participant compliance with instructions.
- **Location of Data Experimenter During Experiment:** In a separate control room or outside the testing booth for the entire experiment.
- **Experiment Script to be Used:** Presentation.

III. Audio Delivery During Experiment:

- **Training Experimenter:** Headphones (Beyerdynamics DT770 PRO; experimenter removed these during the test trials).
- **Participant:** Ear inserts (Etymotic ER2; dB were not measured).

IV. Changes in Pre-registration Due to COVID Pandemic:

- **Data collection completed:** June 12th, 2019.

The lab's pre-registration was followed in all other respects.

Lab 9: mindlab-neu

Contributing Authors: Psyche Loui[1], Parker Tichko[1]
[1] Department of Music, Northeastern University, Boston, MA, United States

Funding Acknowledgements: NIH R21AH075232, NIH R01AG078376, NSF-CAREER 1945436, NSF-BCS 2240330

Pre-Registration

I. Participants:

- **Stopping Criteria:** When I have tested the specific number of participants listed below, accounting for exclusions prior to looking at the data.
- **Sample Size Commitment:** Full block of 16, and we will stop after we reach 16.
- **Compensation:** Financial compensation ($15/hour).
- **Normal Hearing:** Relied on self-report only.

II. Experiment Set-Up:

- **Testing Language:** English.
- **Experiment Room:** Sound -“proof” booth (single walled).

bioRxiv preprint doi: https://doi.org/10.1101/2025.03.13.643168; this version posted March 17, 2025. The copyright holder for this preprint (which was not certified by peer review) is the author/funder, who has granted bioRxiv a license to display the preprint in perpetuity. It is made available under aCC-BY 4.0 International license.

- **Location of Training Experimenter During Experiment:** In the same room/booth as the participant, and they will remain there throughout the experiment to ensure participant compliance with instructions.
- **Location of Data Experimenter During Experiment:** In a separate control room or outside the testing booth for the entire experiment.
- **Experiment Script to be Used:** Psychopy.

III. Audio Delivery During Experiment:

- **Training Experimenter:** Headphones (Sennheiser 280; experimenter removed these during the test trials).
- **Participant:** Headphones (Sennheiser 280; dB were not measured).

IV. Changes in Pre-registration Due to COVID Pandemic:

- **Data collection completed:** November 2nd, 2021.

The lab's pre-registration was followed in all other respects.

Lab 10: bdl_uakl

Contributing Authors: Grace A. Lourie[1], David Moreau[1]
[1] School of Psychology and Centre for Brain Research, University of Auckland, Auckland, New Zealand

Funding Acknowledgements: None

Pre-Registration

I. Participants:

- **Stopping Criteria:** When I have tested the specific number of participants listed below, accounting for exclusions prior to looking at the data.
- **Sample Size Commitment:** Half block of 8, but will continue collecting until the end date for data collection.
- **Compensation:**
- **Normal Hearing:** Conducted hearing tests (KUDUwave Software or equivalent).

II. Experiment Set-Up:

- **Testing Language:** English
- **Experiment Room:** Sound -"proof" booth (single walled).

bioRxiv preprint doi: https://doi.org/10.1101/2025.03.13.643168; this version posted March 17, 2025. The copyright holder for this preprint (which was not certified by peer review) is the author/funder, who has granted bioRxiv a license to display the preprint in perpetuity. It is made available under aCC-BY 4.0 International license.

- **Location of Training Experimenter During Experiment:** In the same room/booth as the participant, and they will remain there throughout the experiment to ensure participant compliance with instructions.
- **Location of Data Experimenter During Experiment:** In a separate control room or outside the testing booth for the entire experiment.
- **Experiment Script to be Used:** Psychtoolbox.

III. Audio Delivery During Experiment:

- **Training Experimenter:** Headphones (Etimotic ER3; experimenter removed these during the test trials).
- **Participant:** Headphones (Etimotic ER3; dB were measured; 23 dB in silence, 26 dB to sound (headphones in contact with decibel meter)).

IV. Changes in Pre-registration Due to COVID Pandemic:

- **Data collection completed:** March 5th, 2020.
- **Compensation:** Participants were compensated with either a voucher ($10/hour) or course credits (0.5 units/half hour), if they were recruited from UofA Experiential Learning Components (PSYCH 204 and PSYCH 207).

The lab's pre-registration was followed in all other respects.

Lab 11: smartlab-ryerson

Contributing Authors: Sean A. Gilmore[1], Frank A. Russo[1]
[1] Department of Psychology, Toronto Metropolitan University, Toronto, ON, Canada

Funding Acknowledgements: Natural Science and Engineering Research Council (2017-06969)

Pre-Registration

I. Participants:

- **Stopping Criteria:** When I have tested the specific number of participants listed below, accounting for exclusions prior to looking at the data.
- **Sample Size Commitment:** Full block of 16, and will stop after we reach 16.
- **Compensation:** Course credit (2 ½ credits).
- **Normal Hearing:** Relied on self-report only.

II. Experiment Set-Up:

- **Testing Language:** English.

bioRxiv preprint doi: https://doi.org/10.1101/2025.03.13.643168; this version posted March 17, 2025. The copyright holder for this preprint (which was not certified by peer review) is the author/funder, who has granted bioRxiv a license to display the preprint in perpetuity. It is made available under aCC-BY 4.0 International license.

- **Experiment Room:** Sound-“proof” booth (single walled).
- **Location of Training Experimenter During Experiment:** In the same room/booth as the participant, and they will remain there throughout the experiment to ensure participant compliance with instructions.
- **Location of Data Experimenter During Experiment:** In a separate control room or outside the testing booth for the entire experiment.
- **Experiment Script to be Used:** Presentation.

III. Audio Delivery During Experiment:

- **Training Experimenter:** Ear inserts (3M E-A-RTONE Gold 3A; experimenter removed these during test trials).
- **Participant:** Ear inserts (3M E-A-RTONE Gold 3A; dB were not measured).

IV. Changes in Pre-registration Due to COVID Pandemic:

- **Data collection completed:** July 5th, 2022.

The lab’s pre-registration was followed in all other respects.

Lab 12: unlv-acnl

Authors: Karli M. Nave[1,2], Joel S. Snyder[1], Erin E. Hannon[1]
[1] Department of Psychology, University of Nevada Las Vegas, Las Vegas, NV, United States
[2] Department of Psychology, Centre for Brain and Mind, University of Western Ontario, London, ON, Canada

Funding Acknowledgements: UNLV Barrick Fellowship (2018-2019, to KMN), UNLV Summer Doctoral Fellowship (2019 & 2020, to KMN), UNLV Top Tier Doctoral Graduate Research Assistantship (2019-2021, to EEH)

Pre-registration

I. Participants:

- **Stopping Criteria:** Data collection deadline (will continue testing as many participants as possible before that date).
- **Sample Size Commitment:** Full block of 16, then will continue collecting until the end date for data collection.
- **Compensation:** Course credit (1 credit per hour).
- **Normal Hearing:** Verified via a hearing (audiometer) test.

II. Experiment Set-Up:

bioRxiv preprint doi: https://doi.org/10.1101/2025.03.13.643168; this version posted March 17, 2025. The copyright holder for this preprint (which was not certified by peer review) is the author/funder, who has granted bioRxiv a license to display the preprint in perpetuity. It is made available under aCC-BY 4.0 International license.

- **Testing Language:** English.
- **Experiment Room:** Sound-attenuated booth (single-walled).
- **Location of Training Experimenter During Experiment:** In the same room/booth as the participant during the entire study.
- **Location of Data Experimenter During Experiment:** In a separate area/room outside of the testing booth during the entire study.
- **Experiment Script to be Used:** Presentation script* (written by K. Nave).
  *Default script provided for use by participating labs.

III. Audio Delivery During Experiment:

- **Training Experimenter:** Sennheiser 280 Pro headphones (experimenter removed these during test trials).
- **Participant:** Ear inserts (Etymotic ER-3; dB levels measured: 20 dB in silence, 60 dB to sound).

IV. Changes in Pre-registration Due to COVID Pandemic:

- **Data collection completed:** September 20th, 2021.

The lab's pre-registration was followed in all other respects.

Lab 13: UoD-keitellab

Contributing Authors: Sarah C. Allen[1], Anne Keitel[1]
[1] Department of Psychology, University of Dundee, Dundee, UK

Funding acknowledgements: Medical Research Council (Grant Number MR/W02912X/1; RSE Saltire Facilitation Network Award (Reference Number 1963)

Pre-Registration

I. Participants:

- **Stopping Criteria:** When I have tested the specific number of participants listed below, accounting for exclusions prior to looking at the data
- **Sample Size Commitment:** Full block of 16, and will stop after we reach 16
- **Compensation:** Financial compensation (Â£15)
- **Normal Hearing:** Relied on self-report only

II. Experiment Set-Up:

- **Testing Language:** English

bioRxiv preprint doi: https://doi.org/10.1101/2025.03.13.643168; this version posted March 17, 2025. The copyright holder for this preprint (which was not certified by peer review) is the author/funder, who has granted bioRxiv a license to display the preprint in perpetuity. It is made available under aCC-BY 4.0 International license.

- **Experiment Room:** Room with occasional mild/moderate noise from adjacent room(s)
- **Location of Training Experimenter During Experiment:** In the same room/booth as the participant, and they will remain there throughout the experiment to ensure participant compliance with instructions
- **Location of Data Experimenter During Experiment:** In a separate control room or outside the testing booth for the entire experiment
- **Experiment Script to be Used:** Psychtoolbox

III. Audio Delivery During Experiment:

- **Training Experimenter:** Headphones (Diablo Draco HS880; experimenter removed these during the test trials)
- **Participant:** Headphones (Sennheiser HD 25; dB were measured; ~39 dB in silence, ~46 dB to sound)

IV. Changes in Pre-registration Due to COVID Pandemic:

- **Data collection completed:** December 2nd, 2021
- Location of Training Experimenter during Experiment: The experimenter left the room during the experiment phase
- **The lab's pre-registration was followed in all other respects.**

Lab 14: vumc-mtsu

Contributing Authors: Reyna L. Gordon[1], Eniko Ladanyi[1,2], Cyrille L. Magne[3], Srishti Nayak[1,2]
[1] Department of Otolaryngology - Head and Neck Surgery, Vanderbilt University Medical Center, Nashville, TN, United States
[2] Department of Linguistics, University of Potsdam, Potsdam, Germany
[3] Department of Psychology, Middle Tennessee State University, Murfreesboro, TN, United States

Funding acknowledgements: NIH DP2HD098859, NSF 1926794, NSF 1926736

Pre-Registration

I. Participants:

- **Stopping Criteria:** When I have tested the specific number of participants listed below, accounting for exclusions prior to looking at the data
- **Sample Size Commitment:** Half block of 8, and will stop after we reach 8

bioRxiv preprint doi: https://doi.org/10.1101/2025.03.13.643168; this version posted March 17, 2025. The copyright holder for this preprint (which was not certified by peer review) is the author/funder, who has granted bioRxiv a license to display the preprint in perpetuity. It is made available under aCC-BY 4.0 International license.

- **Compensation:** Financial compensation ($20)
- **Normal Hearing:** Used an audiometer to conduct hearing tests

II. Experiment Set-Up:

- **Testing Language:** English
- **Experiment Room:** Sound-“proof” booth (double walled)
- **Location of Training Experimenter During Experiment:** The experimenter will leave the booth during the testing phase and the participant will be monitored through a camera from outside
- **Location of Data Experimenter During Experiment:** In a separate control room or outside the testing booth for the entire experiment
- **Experiment Script to be Used:** E-Prime 2.0

III. Audio Delivery During Experiment:

- **Training Experimenter:** Headphones (Sony WH-1000X MH; experimenter removed these during the test trials)
- **Participant:** Headphones (Bose QuietComfort 35; dB was not measured)

IV. Changes in Pre-registration Due to COVID Pandemic:

- **Data collection completed:** April 26th, 2022

The lab’s pre-registration was followed in all other respects.
